# Utility of mosquito surveillance data for spatial prioritization of vector control against dengue viruses in three Brazilian cities

**DOI:** 10.1186/s13071-015-0659-y

**Published:** 2015-02-15

**Authors:** Kim M Pepin, Clint B Leach, Cecilia Marques-Toledo, Karla H Laass, Kelly S Paixao, Angela D Luis, David TS Hayman, Nels G Johnson, Michael G Buhnerkempe, Scott Carver, Daniel A Grear, Kimberly Tsao, Alvaro E Eiras, Colleen T Webb

**Affiliations:** Fogarty International Center, National Institute of Health, Bethesda, Maryland 20892 USA; United States Department of Agriculture, National Wildlife Research Center, Wildlife Services, Animal and Plant Health Inspection Service, 4101 Laporte Ave, Fort Collins, CO 80521 USA; Department of Biology, Colorado State University, Fort Collins, Colorado 80523 USA; Ecovec S.A, Belo Horizonte, Minas Gerais Brazil; Departamento de Parasitologia, Universidade Federal de Minas Gerais, Av. Pres. Antonio Carlos, 6627, Pampulha, Belo Horizonte, MG Brazil; Department of Biology, University of Florida, Gainesville, Florida 32611 USA; School of Biological Sciences, University of Tasmania, Hobart, 7000 Australia; Current address: Department of Wildlife Biology, College of Forestry and Conservation, University of Montana, Missoula, Montana 59812 USA; Current address: Department of Ecology and Evolutionary Biology, University of California – Los Angeles, Los Angeles, California 90095 USA; Current address: EpiLab, Infectious Disease research Centre (IDReC), Hopkirk Research Institute, Institute of Veterinary, Animal and Biomedical Sciences, Massey University, Palmerston North, Manawatu New Zealand

**Keywords:** Vector control, Dengue, Surveillance, Vector density, Mosquito-human interactions, Gravity model, INLA

## Abstract

**Background:**

Vector control remains the primary defense against dengue fever. Its success relies on the assumption that vector density is related to disease transmission. Two operational issues include the amount by which mosquito density should be reduced to minimize transmission and the spatio-temporal allotment of resources needed to reduce mosquito density in a cost-effective manner. Recently, a novel technology, MI-Dengue, was implemented city-wide in several Brazilian cities to provide real-time mosquito surveillance data for spatial prioritization of vector control resources. We sought to understand the role of city-wide mosquito density data in predicting disease incidence in order to provide guidance for prioritization of vector control work.

**Methods:**

We used hierarchical Bayesian regression modeling to examine the role of city-wide vector surveillance data in predicting human cases of dengue fever in space and time. We used four years of weekly surveillance data from Vitoria city, Brazil, to identify the best model structure. We tested effects of vector density, lagged case data and spatial connectivity. We investigated the generality of the best model using an additional year of data from Vitoria and two years of data from other Brazilian cities: Governador Valadares and Sete Lagoas.

**Results:**

We found that city-wide, neighborhood-level averages of household vector density were a poor predictor of dengue-fever cases in the absence of accounting for interactions with human cases. Effects of city-wide spatial patterns were stronger than within-neighborhood or nearest-neighborhood effects. Readily available proxies of spatial relationships between human cases, such as economic status, population density or between-neighborhood roadway distance, did not explain spatial patterns in cases better than unweighted global effects.

**Conclusions:**

For spatial prioritization of vector controls, city-wide spatial effects should be given more weight than within-neighborhood or nearest-neighborhood connections, in order to minimize city-wide cases of dengue fever. More research is needed to determine which data could best inform city-wide connectivity. Once these data become available, MI-dengue may be even more effective if vector control is spatially prioritized by considering city-wide connectivity between cases together with information on the location of mosquito density and infected mosquitos.

**Electronic supplementary material:**

The online version of this article (doi:10.1186/s13071-015-0659-y) contains supplementary material, which is available to authorized users.

## Background

Understanding the relationship between *Aedes aegypti* vectors and the patterns of dengue fever they cause is important in the design of vector-based disease control strategies. Because it is often not feasible or possible to eradicate the mosquito vectors [1], quantitative knowledge of how vector density relates to disease incidence is essential for deciding how much vector populations need to be reduced in order to decrease disease incidence adequately. Mechanistic knowledge of transmission is also important because methods of vector control that are designed based on perceived spatial patterns of cases are often not effective [1,2]. Identifying how vectors are connected to disease incidence in space and time would allow for more cost-effective strategies of implementing vector controls.

The strength and direction of the relationship between mosquito density and dengue infection varies depending on the spatial scale at which data are collected and community characteristics [3-8]. For example, when comparing adult vector densities with prevalence of human infections across three sets of community conditions (urban, suburban, slum) within Rio de Janeiro, Brazil, Honorio et al. [3] found higher infection prevalence in the slum where vector density was lowest. This negative relationship was hypothesized to be because living conditions in the slum facilitated greater rates of vector-human contact relative to the highly developed urban area. At the household scale, no relationship between vector density and disease prevalence was found [3], although it was acknowledged that larger numbers of infections are required at this scale before appropriate conclusions can be drawn. On the other hand, in rural villages in Thailand, a non-significant but positive trend in the relationship between adult vector density and child infection prevalence was found at the household and between-house levels [7]. Considering that within- and between-house transmission have been shown to be important [9], the weak relationship between adult vector density and human infections at the household level is surprising.

One potential explanation for the weak relationship is sampling – the number of replicate samples in space and time, or techniques used for vector collection, may not be adequate for estimating household mosquito density at a level of precision that is smaller than the ecologically-determined variation in vector density. A second explanation could be human movement [10] – human contact patterns at different spatial scales (local and long-distance) can explain spatial dengue transmission [9,11], highlighting that movement at multiple spatial scales is important to consider when linking vector densities to human cases. Theoretical work has demonstrated that the rate of within-city transmission of dengue virus depends on the type of human movements: regular movement patterns due to commuting patterns, for example, can slow the rate of disease spread by up to 25% in comparison to random movement patterns [12]. In contrast, temporally unstructured movements, such as those found in resource-poor settings, can increase the size of an epidemic by up to 20% [13]. Thus, consideration of human movement at different spatial scales is important for understanding how mosquito density data can be used for targeting vector controls.

Several cities in Brazil have implemented a city-wide mosquito trapping system, MI-Dengue, which monitors weekly prevalence of gravid *Ae. aegypti* and *Ae. Albopictus* city-wide in real time [14-16]. Traps are associated with households and spaced in a grid-like manner at ~200-300 m intervals, depending on the city. Vector density data are automatically available for control personnel who respond by focusing source reduction, larvicide and, more rarely, adulticide activities to neighborhood blocks with high mosquito density. The MI-dengue system – based mainly on the idea that spatially targeting areas with higher densities of gravid female mosquitos will decrease case loads using fewer resources – has been shown to be effective and cost-effective for reducing human infections [16]. It has been demonstrated that confirmed cases in humans cluster with high mosquito density in space and time [14], but rigorous quantitative analyses that identify how to best use the surveillance data have not been conducted. Although information on infected mosquitos and confirmed cases in humans are given the highest weight in spatial prioritization of vector control, these data are rarer and often not available until well after transmission has occurred, emphasizing the importance of identifying the best method of using mosquito density data in spatial prioritization of vector control.

While experiments to determine appropriate spatial scales for estimating vector density are still ongoing, the available data are numerous (~5,726 - 43,467 mosquitoes surveyed annually per city) and could reveal useful insight on the spatio-temporal relationship between vector densities and human cases within entire cities. Here, we sought to better understand the city-wide relationship between vector densities and human cases to provide further guidance for spatially targeting vector control work. Our analysis has the following four aims, to: 1) quantify the relative role of city-wide mosquito surveillance data in predicting city-wide cases of dengue, 2) identify the spatial scale at which case data from other neighborhoods are important, 3) identify whether readily available data related to urban characteristics can be used to approximate spatial patterns of human cases, and 4) understand how city-wide mosquito surveillance data can be used to spatially prioritize vector control activities in order to have the maximum effect on preventing cases of dengue fever. We base our analyses on data from Vitoria city, Brazil, because it had the longest time series of surveillance data (~5 years), but we use data from two other cities for validation of model structure and a deeper understanding of model parameters.

## Methods

### Study site

Models were developed using data from Vitoria city, Brazil, an economically prosperous coastal city that is the largest city (348,265 inhabitants) in the state of Espirito Santo in southeastern Brazil. Among the 27 major cities in Brazil, Vitoria has the 4^th^ highest human development index (HDI; 0.85), the highest gross domestic product per capita and an unemployment rate of 7.25% (Brazilian Institute of Statistics and Geography, 2010 Census). The climate is tropical with an annual mean temperature of 23°C and a rainy season between October and January (National Institute of Meteorology, Brazil). Due to its prosperity, size and port capabilities, there is frequent movement of people and merchandise to and from nearby and more distant cities that are less developed.

Data from two other cities, Governador Valadares (GV; population 263,594) and Sete Lagoas (SL; population 208,847), both in the state of Minas Gerais, Brazil, were used for model testing and validation. Both cities have a history of dengue fever outbreaks and are similarly economically prosperous with HDI and unemployment rates of 0.77 and 6.8% (GV), and 0.76 and 6.8% (SL) (Brazilian Institute of Statistics and Geography, 2010 Census). The river Doce bisects GV acting as a gateway between major marine ports. Annual mean temperatures are 24.6°C (GV) and 20.9°C (SL), with a rainy season between October and March (National Institute of Meteorology, Brazil).

Neighborhood-level population sizes, areas (Additional file 1, spreadsheet "Neighborhoods") and economic data were obtained from the 2010 census (mentioned above), from the local vector control managers and the Ministry of Health Secretaries. For Vitoria, economic values were the sum of the registered commercial (including industry and service) units for each neighborhood. For GV and SL, neighborhood economic data were either the number of registered residences or commercial units per neighborhood.

### Case data

Notified cases of dengue fever were obtained from each city’s Ministry of Health Secretary’s official database, which lists dengue-fever cases by their residential address and date of first symptoms. In Brazil, dengue is a mandatory notifiable disease and thus the database represents all cases where any kind of medical care was sought. However, only samples at the start of an epidemic are validated for the presence of dengue virus. Once an epidemic is deemed started, most other cases are diagnosed symptomatically, such that consistent serotype information is unavailable. Although neighborhood assignments were complete, street address information was often lacking, thus we aggregated the case data to the neighborhood level - a political boundary defined by the city. The numbers of neighborhoods in each city were: Vitoria – 75, GV– 65, SL – 98. Neighborhood population sizes and areas were variable both within and between cities (mean ± 2 standard errors for population sizes and areas in km^2^ were: Vitoria – 4,080 ± 1,614, 0.47 ± 0.11; GV – 3,435 ± 862, 3.14 ± 4.69; SL – 2,000 ± 327, 0.37 ± 0.06; Additional file 1: Table S1). We summed the cases in weekly intervals to match the temporal scale of the mosquito data.

### Mosquito surveillance data

Mosquito data were obtained from a city-wide surveillance system (MI-Dengue) [15] managed by the company, Ecovec, which originated from an academic setting and is located in Belo Horizonte, MG, Brazil. The system is comprised of a network of sticky traps, called MosquiTRAP, which have been extensively tested and described elsewhere [17-21]. Briefly, traps are placed in a lattice throughout the entire city. Each trap is checked weekly for mosquitos, which are identified to species level. The data are entered by cell phones to a database that automatically generates maps of mosquito density for control personnel, who target control to highly infested areas. We obtained weekly counts of the gravid female *Ae. aegypti* (93.2– 98.4% of all mosquitos depending on city) and *Ae. albopictus* species, the primary and secondary vectors of dengue fever. Because each trap was located on the inside or outside of a residence, we expressed the mosquito data as average household mosquito density per neighborhood (mosquitos/traps per neighborhood per week; 18.6 traps/neighborhood on average) to match the spatial scale of the available case data. Using an average household abundance estimate also has the advantage of reducing the uncertainty in household mosquito density compared with using single-replicate trap-level counts for each time point. Mean number of mosquitos and traps counted per week across the three cities were: Vitoria – 716.8 ± 342.5 standard deviation (SD) and 1391.6 ± 32.0 SD, respectively; GV – 212.5 ± 81.8 SD and 373.0 ± 50.4 SD, respectively; SL – 95.4 ± 72.3 SD and 411.2 ± 123.8 SD, respectively (Table S1).The area monitored per city was: 33 km^2^ (Vitoria), 27 km^2^ (GV) and 31 km^2^ (SL), which yields mean weekly trap monitoring densities of 42.2, 13.8 and 13.3 traps per km^2^ in the three cities respectively (Additional file 1, Spreadsheet "Traps").

In all three cities, routine vector control occurs following guidelines of the Brazilian Dengue Control Program. This includes mainly larvacide and source reduction activities that occur systematically (moving from block to block) throughout each city year-round. In addition to these activities, adulticide is conducted in blocks where high numbers of mosquitoes are identified, following the recommendations by Ecovec (www.ecovec.com). The effects of these controls, and other factors that affect mosquito populations such as weather, are implicit in the mosquito density data. Thus, although mosquito populations are altered by several biotic and abiotic factors, the mosquito surveillance data are a means of directly examining effects of mosquito density on disease incidence.

### Statistical model structure and parameter estimation

Weekly cases of dengue fever in each neighborhood in Vitoria from Nov. 2007 through Dec. 2011 (4.17 years) were used first for model fitting. Data were modeled using a generalized linear mixed model with a Poisson error structure and log link. Differences between neighborhoods in population size are accounted for through an offset term. Random effects of neighborhood were included to account for within-neighborhood error correlations. The full model used for model selection was of the form:

1 $$ \begin{array}{l}y\left(i,t\right)\sim Poisson\left[\lambda \left(i,t\right)\right],\\ {}\lambda \left(i,t\right)= \exp \left[Y\left(i,t\right)+ \log \left(P(i)\right)+\pi (i)\right],\\ {}\pi (i)\sim N\left[0,{\sigma}^2\right],\end{array} $$

where *Y*(*i,t*) is defined in Eqn. 3 (below), *P* is the neighborhood population size and *π* is the random effect of neighborhood*.* In order to compare the role of mosquito density data in prediction of case notifications at a larger spatial scale, an analogous general linear model with mosquito covariate data aggregated to the city level was analyzed. Note that in this model structure, connectivity between neighborhoods, random effects of neighborhoods and differences in neighborhood population sizes were irrelevant and thus the model structure reduces to a simple linear regression with a Poisson error structure as follows:

2 $$ \begin{array}{l}y(t)\sim Poisson\left[\lambda (t)\right],\\ {}\lambda (t)= \exp \left[Y(t)\right],\end{array} $$

where *Y*(*t*) represents mean mosquito density in the entire city during week *t*. Approximate Bayesian inference by integrated nested Laplace approximations was used for parameter estimation. R software Version 3.0.1 and the package R-INLA (www.r-inla.org) were used to perform the analyses [22].

### Description of covariates

The importance and structure of spatial coupling between neighborhoods (a proxy for human movement) was examined as a main effect using a modified gravity model (described below). All covariate data were normalized in order to compare the strength of parameter estimates. A term for spatial autocorrelation was not included in the final models because it was not significant (according to a Moran’s I test on shifted residuals) in preliminary fitting of gravity model terms. We also compared our models, which included a covariate-based exploration of the case data, with autoregressive lag 1 models (AR1) and found similar levels of predictive power (data not shown).

Gravity models have been used effectively to explain the spatial spread of measles between cities in England [23]. The traditional gravity model assumes that movement between locations is a function of both population size and distance between populations. The concept is that areas with large population sizes act as disease sources by attracting susceptible hosts. The “force” of disease spread becomes less strong the further away hosts are from the large populations. This relationship works well for describing infection spread *between* cities [23,24], but human movement between neighborhoods within a city due to commuting, visiting friends or going to shopping areas [9,10] may not necessarily be correlated with population size and/or distance. Secondly, dengue is a vector-borne disease, meaning that the presence of vectors is required for transmission from the donor population. Thus, we used a modified version of a gravity model, incorporating effects of mosquito density and using additional neighborhood characteristics to describe spatial coupling. We were interested in testing whether these commonly available approximations could be useful for interpreting mosquito surveillance data in terms of human cases because direct measures of neighborhood connectivity are not usually available without time-consuming, expensive field studies. In the full model, the rate of case notification in neighborhood *i* at time *t* is:

3 $$ {Y}_{i,t}={\beta}_1{M}_{i,t-x1}+{\beta}_2{Y}_{i,t-y1}+{\beta}_3{M}_{i,t-x1}{Y}_{i,t-y1}+{\beta}_4{\sum}_{\mathrm{j}}{M_{j,t-x2}}^{\alpha 1}+{\beta}_5{\sum}_{\mathrm{j}}{\left({Y_{j,t}}_{-y2}/f\left({x}_j\right)\right)}^{\alpha 2}+{\beta}_6\sum \mathrm{j}{\left({M}_{j,t-x2}{Y_{j,t}}_{-y2}/f\left({x}_{\mathrm{j}}\right)\right)}^{\alpha 3}, $$

where *Y* is the number of cases, *M* is the mosquito-trap prevalence, *α* is a scaling parameter, *i* and *j* denote neighborhoods (where *i* ≠ *j*), *t* is the weekly time step, *x1, x2, y1* and *y2* are time lags in weeks for mosquito and disease data at the within and between-neighborhood scales. *f(x*_*j*_*)* is a proxy for neighborhood connectivity (*i.e.*, a term for weighting case notifications in neighborhood *j* according to factors that could describe disease connections between neighborhoods, such human movement; Table 1). Distance was calculated in ArcGIS using road data, such that the distance between neighborhoods was proportional to the amount of travel time between neighborhood centroids (or centroid adjusted to the nearest road). Note that *f(x*_*j*_*)* does not vary in time, which is an appropriate approximation since our time series is <5 years.

**Table 1:** **Candidate structures for the components of the** ***f(x***_***j***_ ***)***

**Components of** ***f(x***_***j***_ ***)***	**Description of hypothesis tested**
1) 1	H_1_: Dengue cases occur in a random spatial pattern based on the number of cases in all other neighborhoods.
2) *d*_*ij*_^*α*^	H_2_: The spatial pattern of dengue-case occurrence correlates with distance between other neighborhoods; cases are more likely to occur in neighborhoods that are closer to neighborhoods experiencing cases.
3) (1/*E* ^*^_j_)^*α*^	H_3_: The spatial pattern of dengue-case occurrence correlates with neighborhood economy values; cases are more likely to occur if neighborhoods with high-economy values are experiencing cases.
4) (1/*D*_*j*_)^*α*^	H_4_: The spatial pattern of dengue-case occurrence correlates with neighborhood population density values; cases are more likely to occur if neighborhoods with high-density values are experiencing cases.

*Note that for GV and SL, there was no economy index as for Vitoria. Thus, the number of commercial buildings and the number of residences in each neighborhood were used separately as comparable economic values.

### Model selection

The criteria used for model selection were Deviance Information Criterion (DIC; [25]) and the mean log Conditional Predictive Ordinates (mlCPO), which is analogous to leave-one-out cross-validation [26]. Lower DIC and mlCPO values indicate better predictive power of the model. Because the mlCPO showed the same rank order as a measure of explained variation (Spearman’s *r* coefficient between the observed and model predicted data), we only present the DIC alongside *r* for simplicity. Due to the complexity with how the covariate data could impact human cases, model selection was conducted in several stages, broadly as follows:

1. *Selection of lags*. For each possible covariate (as shown in Equation 1) individually, we identified the best lag time between it and the response variable (*x1*, *x2*, *y1*, *y2*, *z1* and *z2* in Equation 1). Lags were calculated as a 3-week average because we hypothesized that a window of time in the past may best explain the relationship (preliminary analyses confirmed this hypothesis). The 3-week window was chosen because 2–3 weeks is the combined amount of time from an infectious mosquito bite to a case report, on average [27]. This is simply the combination of average incubation periods in vectors and humans and assumes that an infectious vector would transmit immediately upon becoming infectious. Thus, lag 1 was the average of weeks 1 to 3 in the past. The longest lag we investigated was 18–20 weeks.
2. *Selection of scaling factors*. Similar to previous work [23], we hypothesized a scaling factor on the gravity terms would be important because these covariates described interactions that could be non-linear. Because initial attempts to fit this parameter were unsuccessful due to the effects of its non-linearity on convergence, we identified the best scaling factor (*α1*-*α3* in Eqn. 3) for each possible between-neighborhood covariate (Eqn. 3, last 3 covariates) by fitting models using a range of fixed scaling factors (*α* = 0.001, 0.01, 0.1, 0.5, 1, 2). These values were chosen because they represent a range of biologically realistic functions for the relationship between gravity components (concave-up, concave-down or linear). The lowest value (i.e., 0.0001) was chosen based on convergence to the lowest DIC (representing asymptotic behavior of the best value) and for values above the highest (i.e., 2) the DIC continued to increase in the exponential part of the curve (i.e., values > 2 did not produce good fits).
3. *Mosquito and human case terms*. We compared models with only mosquito density data (*M*_*i,t-x1*_ and ∑_j_*M*_*j,t-x2*_^*α1*^) to those with only human-case notifications (*Y*_*i,t-y1*_ and ∑_j_(*Y*_*j,t*-y2_/*f*(*x*_j_))^*α2*^), and those with both types of covariate data (*i.e.*, Eqn. 3), to investigate the role of mosquito density data.
4. *Spatial scale of between neighborhood interactions.* For the between-neighborhood effects, we compared two scales: 1) nearest-neighbor effects (*i.e.*, local) – where only covariate data from immediately adjacent neighborhoods were used to predict cases and 2) global effects – where data from all other neighborhoods city-wide were used to predict cases.
5. *Proxies describing between-neighborhood weights*. For the global between-neighborhood covariates, we compared different functions for weighting between-neighborhood effects (*f*(*x*_*j*_) in Eqn. 1), including economic value (1/*E*_*j*_), population density (1/*D*_*j*_) and travel distance between neighborhoods (1/*d*_*ij*_; Table 1). We hypothesized that high-economy or high-density neighborhoods would attract more people on a regular basis, creating hubs for disease transmission and spatial spread. Similarly, we hypothesized that disease transmission from other neighborhoods would be more likely between neighborhoods with faster road travel. These ideas are similar to a recent study showing that dengue hotspots occur along major roads and transportation hubs [28]. Because mosquitos rarely travel beyond 200 m [29], which is mainly within a neighborhood, the weightings were only applied to the terms with case notification data, β_5_ and β_6_ (Eqn. 3), and not the global mosquito term, β_4_ (Eqn. 3).

Because Steps 1 and 2 were not the focus of our analysis, results from these analyses are presented in the Supplementary Material (Additional file 2: Figure S1, Additional file 3: Figure S2, Additional file 4: Figure S3, Additional file 5: Figure S4, Additional file 6: Figure S5, Additional file 7: Figure S6). Results from Steps 3–5 are reported in the main text.

### Model evaluation

All steps were conducted using data from Vitoria from week 45 of 2007 through 2011, thus withholding data from 2012 for evaluation of the final model by out-of-sample prediction (*i.e.*, forecasting). As a second means of model validation, we used the best model selected from Vitoria data on data from two other cities: GV and SL. For this, we re-estimated parameters using the best fit Vitoria-derived model structure from our model selection procedure and covariate data from each other city. Again, we only used a portion of the data for parameter estimation and predicted both this in-sample data as well as the remaining (out-of-sample) data. Because the magnitude and direction of parameter values in the three cities were so different, we did not attempt to predict data in the other two cities using parameters estimated from Vitoria covariate data. Instead, we compared the city-specific parameters.

We also conducted Steps 1, 2, 3 and 5 (above) on data from GV and SL in order to evaluate the generality of conclusions drawn based on the Vitoria time series and to gain a better understanding of how the best model may differ due to city-specific circumstances. The latter two cities did not have as much data: GV in-sample – 90 weeks, GV out-of-sample – 30 weeks, SL in-sample – 86 weeks, and SL out-of-sample – 13 weeks. In-sample data were from 2009 and 2010 while out-of-sample data were from 2011.

## Results

### Role of mosquito data

**Figure 1:**
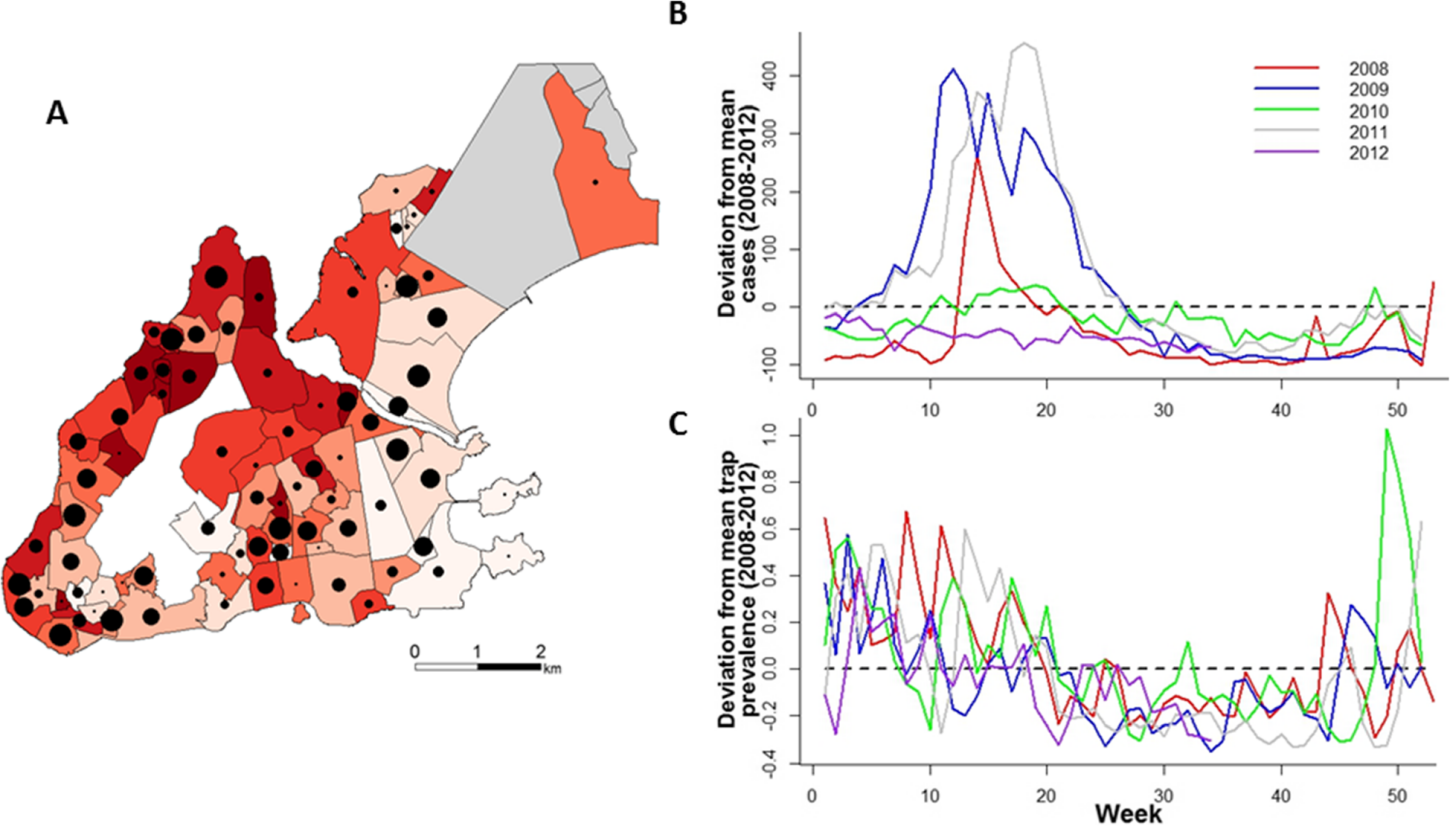
**Trends in mosquito counts and human case data. (A)** Neighborhood distribution of mosquito density (number of mosquitos/number of trap inspections) and prevalence of dengue (number of reported cases/neighborhood population size) between 2008–2012 in Vitoria. Mosquito density is correlated with the size of the black circles, total numbers of cases are indicated by the red shading of neighborhoods (darker color indicates more total cases). The white area in the middle is a steep mountain where no monitoring was conducted. The grey neighborhoods to the north were also not monitored. **(B)** The difference between weekly cases in Vitoria for each year relative to the average number of weekly cases from 2008–2012. Each line represents deviations from a different year as indicated in the legend. **(C)** Same as B but for the prevalence of mosquitoes.

**Figure 2:**
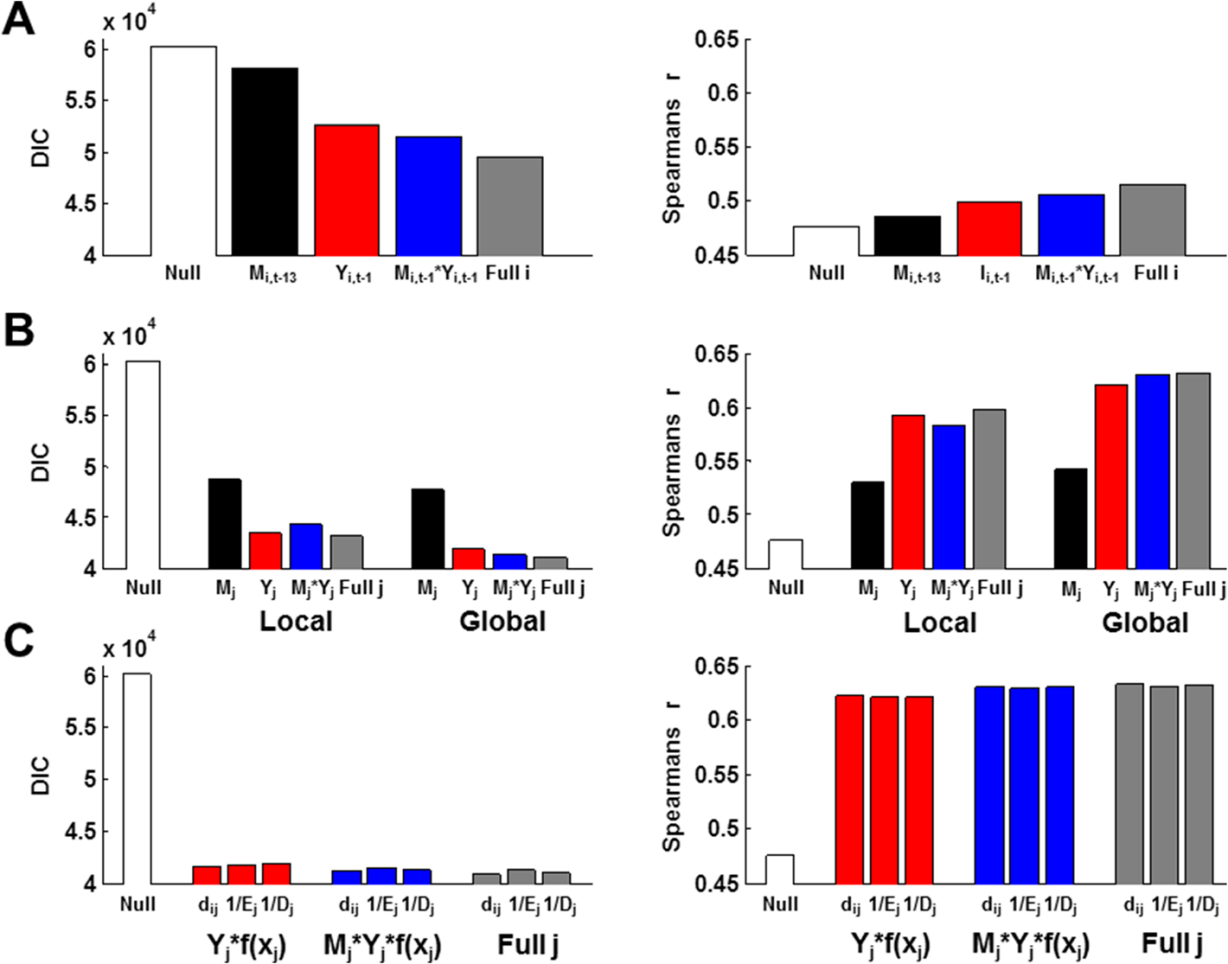
**Model selection results from Vitoria data.** Each bar represents the DIC (left-side plots) or Spearman’s correlation coefficient between the model-predicted and observed data (right-side plots) for a given model. Each covariate was lagged and scaled to the best values (*i.e.*, from model selection shown in Additional file 2: Figure S1 and Additional file 3: Figure S2). “Null” indicates a model with only the neighborhood population size as an offset and random effects of neighborhood. **(A)** Within-neighborhood effects. Lags are indicated. No scaling factors were used. Full *i* is *M*_*i,t-13*_ + *Y*_*i,t-1*_ + *M*_i,t-13_* *Y*_*i,t-1*_. **(B)** Comparison of nearest-neighbor (local) versus all between-neighborhood (global) effects. Lags and scales, respectively, were: 13, 0.5 (local, *Mj*), 12, 0.1 (global, *Mj*), 1, 0.5 (local and global *Yj*), 13 & 1, 0.5 (local *M*_j_*Y_*j*_) and 1 & 1, 0.1 (local *M*_j_*Y_*j*_). Structure of full *j* was *∑*_*j*_ *M*_*j,t-x*_^α1^ + *∑*_*j*_ *Y*_*j, t-y*_^α2^ + *∑*_*j*_(*M*_*j,t-z*_ *Y*_*j,t-y*_) ^α3^. Full *i* covariates (as specified in panel A) were included in each model (*i.e.*, there are 6 covariates in “Full *j*”). **(C)** Effects of the type of approximation (*f(x*_*j*_ *)*) used for weighting global connectivity. Form used for *f(x*_*j*_ *)* is indicated under the bars (*d*, distance; *E*, economy; *D*, density). Full *i* covariates (as specified in panel A) were included in each model (*i.e.*, red and blue bars have 4 covariates). Lags and scales, respectively, were: 1, 0.5 (*Y*_*j*_ *f(x*_*j*_ *)*), 1 & 1, 0.1 (*M*_*j*_ *Y*_*j*_ *f(x*_*j*_ *)*). Structure of full *j* was *∑*_*j*_ *M*_*j,t-12*_^0.1^ + *∑*_j_(Y_*j,t-1*_ *f(x*_*j*_ *)*) ^0.5^ + *∑*_*j*_(*M*_*j,t-1*_ *Y*_*j,t-1*_ *f(x*_*j*_ *)*) ^0.1^.

**Figure 3:**
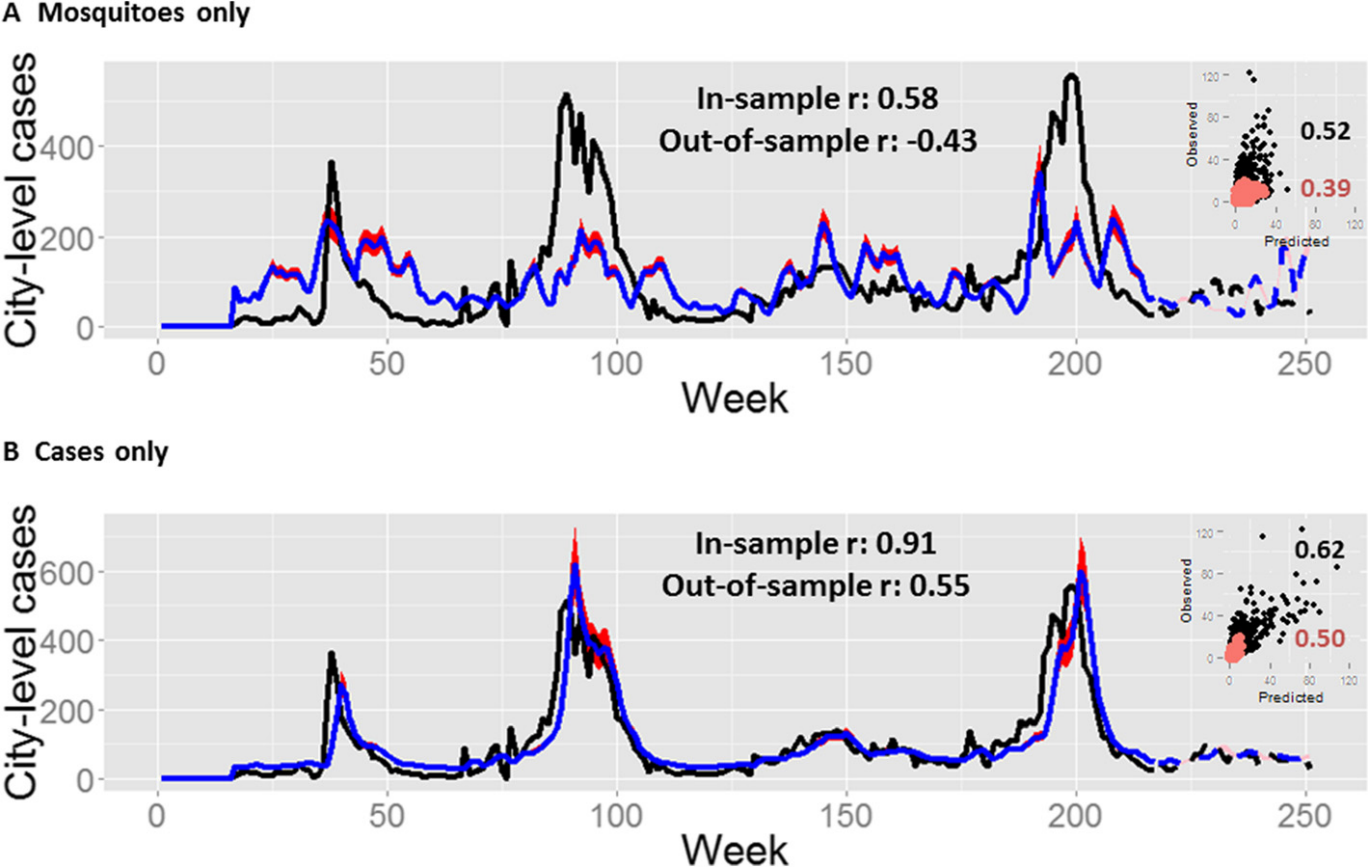
**City-level summary of model fits for Vitoria.** The best model with only mosquito covariates, *M*_*i*_ and *∑*_*j*_ *M*_*j*_, **(A)** is compared to the best model with only dengue-case covariates, *Y*_*i*_ and *∑*_*j*_ *(Y*_*j*_ *f(x*_*j*_ *))* **(B)**. Models were fitted using neighborhood-level data but aggregated to the city level for presentation. Goodness-of-fit was calculated as the Spearman’s correlation (*r*) between the observed and model-predicted values for the fitted model (“In-sample”, solid lines) and out-of-sample predictions (dashed lines). Correlation coefficients are presented for both the aggregated city-level data (main plots) and for the neighborhood-level results presented in the scatterplot insets. Observed data (black lines; solid: in-sample, dashed: out-of sample); model predictions (blue lines; solid: in-sample, dashed: out-of sample), 97.5% credible intervals (red shades: in-sample; pink shades: out-of-sample). **(A)** *Y*_*i,t*_ = *β*_1_ *M*_*i,t-13*_ + *β*_*2*_∑_*j*_ *M*_*j,t-12*_^0.1^ + log(*P*_*i*_) + *π*_*i*_ **(B)** *Y*_*i,t*_ = *β*_1_ *Y*_*i,t-1*_ + *β*_*2*_∑_*j*_(*Y*_*j,t-1*_ */d*_*ij*_ ) ^0.5^ + log(*P*_*i*_) + *π*_*i*_; only the best *Y*_*j*_ term is presented (although they are all similar). For both models, the best lag and scale terms were included as indicated (lag terms are a mean from a 3-week window, *e.g.*, 13 represents the mean for weeks 13–15). *Y* are cases, *M* are mosquitos, *P* is population size, *π* is the neighborhood random effect, *t* is the week, *i* is the target neighborhood and *j* are the sum of all other neighborhoods that are not *i*.

**Figure 4:**
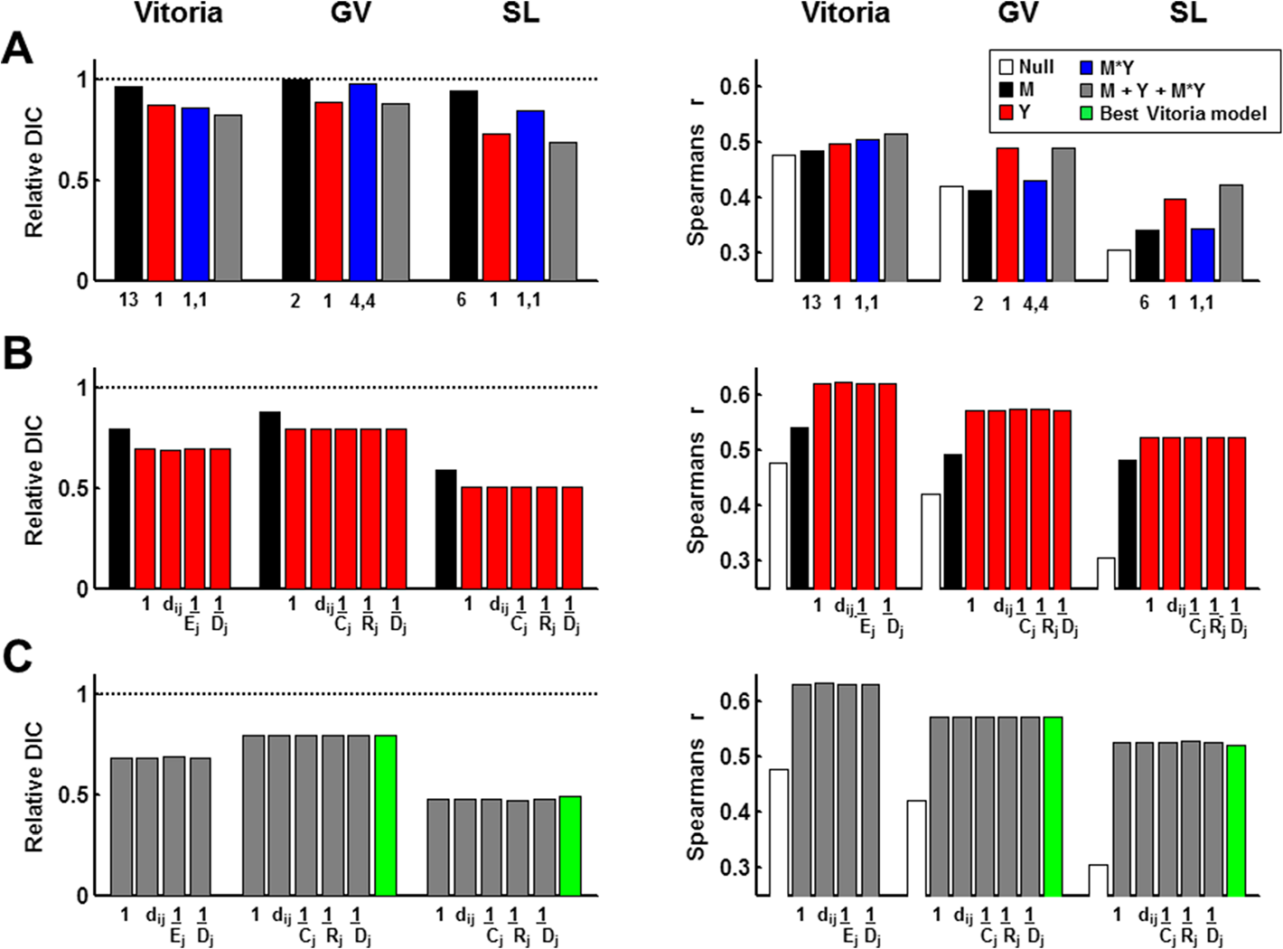
**Comparison of Vitoria model selection results with other cities.** Each bar represents the relative DIC (left-side plots) or Spearman’s correlation coefficient between the model-predicted and observed data (right-side plots) for a given model. Relative DIC is the model DIC divided by the DIC for the null model (model with only the offset and random effects of neighborhood). Each covariate was lagged and scaled using the best values (*i.e.*, from model selection shown in Additional file 2: Figure S1, Additional file 3: Figure S2, Additional file 4: Figure S3, Additional file 5: Figure S4, Additional file 6: Figure S5, Additional file 7: Figure S6). **(A)** Within-neighborhood effects. Lags are indicated beneath the bars. Model structure is shown in the legend. No scaling factors were used. **(B)** Comparison of the type of approximation (*f(x*_*j*_ *)*) used for weighting global connectivity on the case-notification terms (red). Form used for *f(x*_*j*_ *)* is indicated under the bars (1, random; *d*, distance; *E*, economy; C, commercial structures; R, residences; *D*, density). The global mosquito term is shown for comparison (black bar). Full *i* covariates were included in each model (*i.e.*, there are 4 covariates). **(C)** Comparison of *f(x*_*j*_ *)* in the full models. Form used for *f(x*_*j*_ *)* is indicated under the bars (as in B). Full *i* covariates were included in each model (*i.e.*, there are 6 covariates).

There was little visual correlation between weekly time series of mosquito data with human case data when considering the data across space or time (Figure 1). This lack of visual correlation is confirmed using a spatio-temporal Bayesian regression model that accounted for both within- and between-neighborhood effects of mosquito density (Figures 2 and 3). Models that included only lagged case data (without mosquito surveillance data) fit the observed case data much better (Figure 3). Only a very slight gain in fit over cases alone (*r* = 0.62 vs 0.63; Figure 3 and Table 2; for cases alone vs the full model, respectively) was obtained by considering the effects of an interaction between mosquito density and case notifications (Figure 2C, right – compare red bars to blue or grey bars), and this did not translate to increased forecasting ability (*r* = 0.50 vs 0.49; Figure 3 and Table 2; for cases alone vs the full model, respectively). Similarly, the mosquito surveillance data alone were only weak predictors of human cases in the other two cities (Figure 4) as well as at the city-level scale in Vitoria (Additional file 8: Figure S7 and Additional file 9: Figure S8). The difference in *R*^2^ between the city-level (0.18; Additional file 9: Figure S8B) compared with the neighborhood-level (0.27; from *r* = 0.52; Figure 3A) spatial scale, highlights that accounting for neighborhood-level effects is important for linking mosquito density to case data.

**Table 2:** **Goodness-of-fit for the best model selected using data from Vitoria**

**City**	**In-sample**			**Out-of-sample**		
	**Neigh.**	**City**	**Weeks**	**Neigh.**	**City**	**Weeks**
**Vitoria**	0.63	0.91	217	0.49	0.30	34
**GV**	0.58	0.91	90	0.49	0.63	30
**SL**	0.51	0.86	86	0.33	0.70	16

For each city, the model was fit to the data using the “in-sample” portion. The estimated parameters were then used to forecast the remaining data (“out-of-sample” portion). Goodness-of-fit was assessed as the Spearman’s correlation coefficient between the observed and predicted data at both the neighborhood and city levels for each portion of predicted data.

### Utility of proxies for weighting between-neighborhood case data

To investigate how spatial dimensions may shape the relationship between mosquito density and human cases of dengue, we included different scales of spatial disease data (local versus global) at the neighborhood-level within Vitoria. The models that included global coupling performed better than those that only allowed for nearest-neighbor connections (Figure 2B). We also considered different factors that could explain patterns of city-wide human movement such as economic value of neighborhoods, population density or distance between them. The best neighborhood-level model for Vitoria was:

$$ log\left({Y}_{i,t}\right)={\beta}_1{M}_{i,t-13}+{\beta}_2{Y}_{i,t-1}+{\beta}_3{M}_{i,t-1}{Y}_{i,t-1}+{\beta}_4{\sum}_{\mathrm{j}}{M_{j,t-5}}^{0.1}+{\beta}_5{\sum}_{\mathrm{j}}{\left({Y}_{j,t-1}/{d}_{ij}\right)}^{0.5}+{\beta}_6{\sum}_{\mathrm{j}}{\left({M}_{j,t-1}{Y_{j,t}}_{-1}/{d}_{ij}\right)}^{0.1} + log\left({P}_i\right)+{\pi_i}_{.} $$

Although the DIC score was lowest for this full, “best” model, the mlCPO’s (data not shown) and *r* values were very similar for all proxies of neighborhood connectivity (Figure 2C). Thus, although the mlCPO’s and *r* values followed the same rank order as the DIC values, the high similarity of *r* values from models with different proxies for neighborhood connectivity did not indicate biologically important differences between the models in any of the cities (Figure 4). In summary, we found that models including global between-neighborhood effects in addition to within-neighborhood effects performed best and that all 3 types of covariates (mosquito density, case notifications and the interaction of these two covariates), but specific proxies for weighting global connectivity were similar to one another.

### Generality of the vitoria model

**Figure 5:**
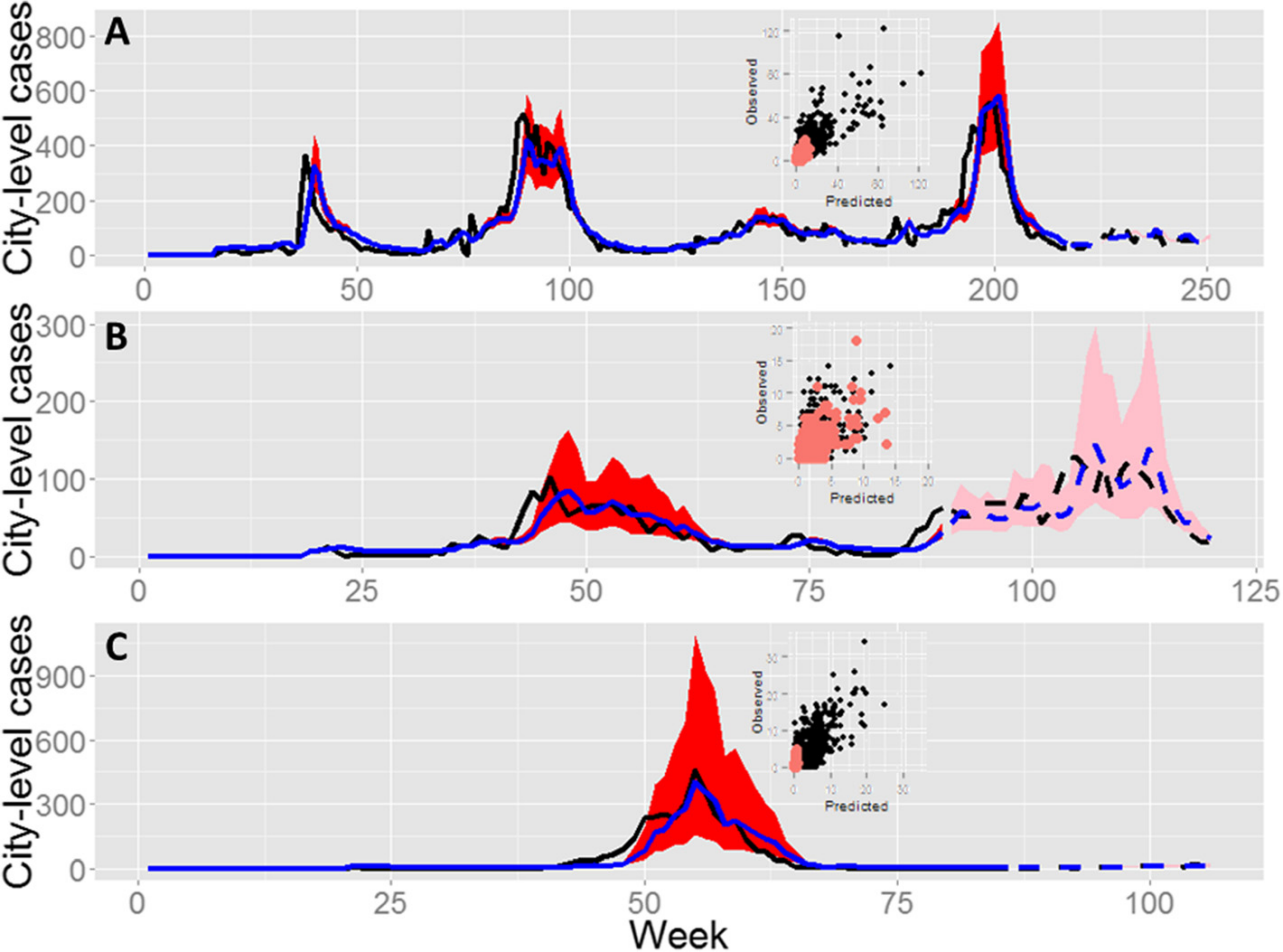
**City-level summary of full-model fits for three cities.** Models were fitted using neighborhood-level data but aggregated to the city level for presentation. Observed data (black lines; solid: in-sample, dashed: out-of sample); model predictions (blue lines; solid: in-sample, dashed: out-of sample), 97.5% credible intervals (red shades: in-sample; pink shades: out-of-sample). Insets display the neighborhood-level fits (black points: in-sample data; pink points: out-of-sample data). Best full model from each city is presented. **(A)** Vitoria: *Y*_i,t_ = *β*_1_ *M*_*i,t-13*_ + *β*_2_ *Y*_*i,t-1*_ + *β*_3_ *M*_*i,t-1*_ *Y*_*i,t-1*_ + *β*_4_∑_j_ *M*_*j,t-5*_^0.1^ + *β*_5_∑_*j*_(*Y*_*j,t-1*_ */d*_*ij*_) ^0.5^ + *β*_6_∑_j_(*M*_*j,t-1*_ *Y*_*j,t*-1_ */d*_*ij*_) ^0.1^ + log(*P*_*i*_) + *π*_*i*._ **(B)** GV: *Y*_i,t_ = *β*_1_ *M*_*i,t-2*_ + *β*_2_ *Y*_*i,t-1*_ + *β*_3_ *M*_*i,t-4*_ *Y*_*i,t-4*_+ *β*_4_∑_j_ *M*_*j,t-1*_^2^ + *β*_5_∑_*j*_(*Y*_*j,t-1*_ *D*_*j*_) ^0.1^ + *β*_6_∑_j_(*M*_*j,t-1*_ *Y*_*j,t*-1_ *D*_*j*_) ^0.5^ + log(*P*_*i*_) + *π*_*i*._ **(C)** SL: *Y*_i,t_ = *β*_1_ *M*_*i,t-6*_ + *β*_2_ *Y*_*i,t-1*_ + *β*_3_ *M*_*i,t-1*_ *Y*_*i,t-1*_ + *β*_4_∑_j_ *M*_*j,t-6*_^0.1^ + *β*_5_∑_*j*_(*Y*_*j,t-1*_ *R*_*j*_) ^0.001^ + *β*_6_∑_j_(*M*_*j,t-1*_ *Y*_*j,t*-1_ *R*_*j*_) ^0.1^ + log(*P*_*i*_) + *π*_*i*._; notation is as in Figure 4.

The general structure of the Vitoria model (including mosquito lags and scaling factors) fit the neighborhood-level data remarkably well in all three cities when the results were interpreted at the city-level (Figure 5, Table 2). For GV, the model also did very well at forecasting future data using parameters that were estimated on an earlier segment of data (Figure 5, Table 2). In Vitoria and SL, the model performed more poorly at forecasting but the forecasted portion of the time series included only a period of low disease prevalence (thus its ability to forecast an upcoming outbreak is unclear). At the neighborhood level, the model produced smaller differences between the observed and model-predicted values in Vitoria and GV relative to SL (Figure 5 (insets), Table 2). When model selection was conducted on GV and SL, models with different mosquito lags and weighting factors for between-neighborhood connectivity (relative to the Vitoria model) were best when considering DIC (Figure 4C, left – compare green bar to grey bars). However, the difference in explained variation and forecasting capability was almost indistinguishable (Figure 4C, right - compare green bar to grey bars), suggesting that the differences in DIC were not biologically important.

The best model for each city included quite different lag times between mosquito density and cases: 13–15 weeks for Vitoria, 1–3 or 2–4 weeks for GV and 6–8 weeks for SL (Additional file 3: Figure S2, Additional file 5: Figure S4 and Additional file 7: Figure S6). However, a lag of 1–3 weeks was always best for the case notification data.

**Figure 6:**
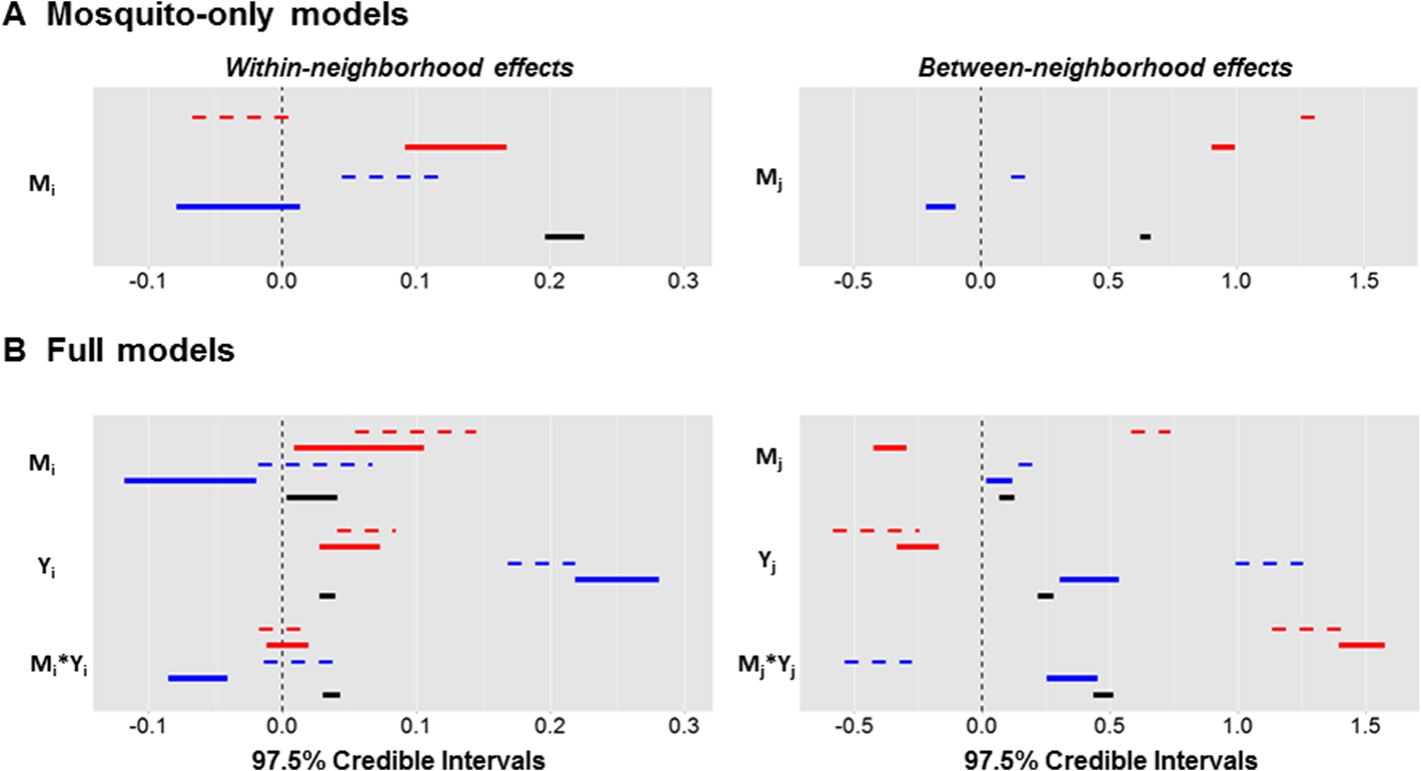
**Credible intervals for each covariate in the mosquitoes-only models (A) and the full models (B).** Vitoria (black), GV (blue), SL (red). Thick solid lines are covariates in the best model selected from Vitoria data: *Y*_i,t_ = *β*_1_ *M*_*i,t-13*_ + *β*_2_ *Y*_*i,t-1*_ + *β*_3_ *Y*_*i,t-1*_ *M*_*i,t-13*_ + *β*_4_∑_j_ *M*_*j,t-5*_^0.1^ + *β*_5_∑_*j*_(*Y*_*j,t-1*_ */d*_*ij*_) ^0.5^ + *β*_6_∑_j_(*M*_*j,t-5*_ * *Y*_*j,t*-1_ */d*_*ij*_) ^0.1^ + log(*P*_*i*_) + *p*_*i*._ Thin dashed lines are for the best models from the other cities: GV: *Y*i,t = *β*_1_ *M*_*i,t-2*_ + *β*_2_ *Y*_*i,t-1*_ + *β*_3_ *M*_*i,t-4*_ *Y*_*i,t-4*_ + *β*_4_∑_j_ *M*_*j,t-1*_^2^ + *β*_5_∑_*j*_(*Y*_*j,t-1*_ *D*_*j*_) ^0.1^ + *β*_6_∑_j_(*M*_*j,t-1*_ *Y*_*j,t*-1_ *D*_*j*_) ^0.5^ + log(*P*_*i*_) + *π*_*i*_; SL: *Y*_i,t_ = *β*_1_ *M*_*i,t-6*_ + *β*_2_ *Y*_*i,t-1*_ + *β*_3_ *M*_*i,t-1*_ *Y*_*i,t-1*_ + *β*_4_∑_j_ *M*_*j,t-6*_^0.1^ + *β*_5_∑_*j*_(*Y*_*j,t-1*_ *R*_*j*_) ^0.001^ + *β*_6_∑_j_(*M*_*j,t-1*_ *Y*_*j,t*-1_ *R*_*j*_) ^0.1^ + log(*P*_*i*_) + *π*_*i*._

In all three cities, between-neighborhood effects were generally stronger than within-neighborhood effects (Figure 6). The strength and direction of mosquito density parameters shifted to some extent when case data were included in the model, although the changes were inconsistent across cities (Figure 6). However, when we compared the parameter values estimated using the Vitoria model to those estimated using the best models from GV and SL, which included different lags for mosquito data (Figure 6, Additional file 10: Figure S9), mosquito parameters show more similar directional effects across the cities. Thus, although the general structure of the Vitoria model may be a useful predictive tool, there are some quantitative differences between cities in the role of mosquito density in predicting cases.

## Discussion

### Role of mosquito data

We found that even with city-wide household-level mosquito surveillance data, the relationship between mosquito density and cases is weak. Although MI-dengue has been effective at decreasing cases city-wide by basing spatial prioritization on within-neighborhood data on mosquito density and recent infections in humans [16], our results highlight that additional data may be useful for further improvements in preventing cases of dengue city-wide. Previous work has similarly found a weak [5-8] or even negative [3,4] relationship between household mosquito density and cases. Part of the reason for the obscured understanding of the role of mosquito densities can likely be attributed to high variation in vector competence across relatively fine spatial and temporal scales [30], emphasizing that surveillance for infected mosquitos should be prioritized. In fact, a strong relationship between the density of infected mosquitos and cases has been observed [8]. In our system, a new technology which monitors the density of infected mosquitoes by serotype, MI-Virus, was recently developed but has not been implemented long enough or city-wide in order for us to have evaluated the data in this study (although the information provided by MI-virus is already being used for spatial targeting of vector control where it is available). As city-wide MI-Virus data become available at the same spatial scale as the density estimates, analyses should be extended to include these data, which may lead to more accurate guidance for spatial prioritization of vector controls. Similarly, to extract the most information from the MI-virus data, it will be important to obtain data on human diagnoses at the level of serotype because the relationship between mosquito density and human cases depends on the interaction of serotype-specific pre-existing immunity and the prevalence of different serotypes [31,32].

Understanding the role of mosquito density in predicting human cases of dengue fever under any experimental design is complicated by sampling scale and variability. It is thought that the mosquito density required to sustain transmission is in fact very low [33]. If the sampling techniques used to enumerate mosquito density are too coarse to distinguish prevalence values around the transmission threshold, then it is possible that a sampling protocol with more replication, or a trapping technology that captures more mosquitos, is required. Studies that aim to determine the precision needed to distinguish low mosquito densities (i.e., near the transmission threshold) with adequate precision, such as mass trapping in enclosed mosquito populations of known sizes using various levels of replication and spatial arrangements, are needed to assess accuracy and precision of mosquito surveillance data. Likewise, better quantification of mosquito thresholds that permit transmission among humans is important for choosing appropriate trapping parameters.

In Brazil, routine vector control occurs city-wide throughout the year following national vector control guidelines [34]. Very broadly, personnel move through entire cities, block by block, neighborhood by neighborhood, in a systematic manner over the course of several months, mainly applying larvicide and source reduction. Documentation of these efforts was too sparse to be included in our models, but we do not expect that they would have obscured our ability to quantify the relationship between mosquito density and cases because they target immature stages and our system quantifies gravid adult females at a weekly scale. Additional controls are spatially targeted based on mosquito surveillance data, dengue cases data and data on infected mosquitos when they are available. However, controls based on human cases are often too late to prevent transmission because suspected cases are not confirmed until 6–8 weeks after notification. In cities where MI-dengue surveillance is conducted, the additional control activities can be targeted to blocks with the highest mosquito densities (or blocks with infected mosquitos where MI-Virus data are available) very rapidly after the mosquito populations achieve high numbers because the longest time lag between trap checking is one week and data can be visualized on the on-line MI-dengue mapping system immediately after a trap is examined [15]. If MI-dengue-based vector-control work varies in intensity non-randomly, as is likely the case due to variability in efficacy that depends on urban structures, and some blocks are responsible for more transmission than others, the combined effect could be a weak relationship between mosquito density and human cases. Moreover, the MI-dengue-based vector-control activities could explain the different best fitting lags for mosquito data among cities, if for example, in some cities the lag between transmission and available data/response is consistently longer than in other cities. Better documentation of the timing, intensity and effectiveness of vector control work in response to MI-dengue surveillance data is needed to investigate how these activities may affect interpretation of how to use mosquito density data for strategic planning of vector control work.

Although models including only mosquito data performed more poorly than those containing only case-notification data, the interaction between mosquito density and case notifications was strongly significant in all three cities. Thus, consideration of the mosquito-human interaction is important in order to more accurately predict cases in space and time. Theoretical work has similarly found a low correlation between *R*_*0*_ (the average number of secondary cases in a naïve population) and mosquito density within an area due to human movement [10]. Also, when the mosquito population is highly heterogeneous, frequent travel to areas with high mosquito density can cause an epidemic or sustain low levels of transmission (depending on connectivity levels) [35], providing mechanistic insight into why mosquito density alone may not be a good predictor of human cases. The importance of between-neighborhood effects in our models suggests that movement among neighborhoods is an important driver of dengue dynamics and that the neighborhood scale, given appropriate movement data, may be effective at capturing mosquito-human interactions.

### Utility of proxies for weighting between-neighborhood case data

We found that global between-neighborhood effects were stronger than either nearest-neighbor effects or within-neighborhood effects, suggesting that many infections occurred distant from the home neighborhood. Our finding that non-local effects within a city impact spatial dynamics is similar to previous work where significant spatio-temporal clustering occurs at distances up to 2.8 km [36] and where 34.7% of cases did not show any spatio-temporal clustering [11]. However, the stronger role of non-local relative to local spatial coupling in our study contrasts the finding that house-to-house human movement may predominantly drive spatial spread [9]. This discrepancy in the relative role case data from further distances may at least partly be due to differences in urban characteristics and human behavior.

While our results show that city-wide cases impact how mosquito density translates to human cases, we were not able to understand its mechanistic nature more fully given the available data. We hypothesized that economic values, population densities or travel time on roads may be good approximations to commuting patterns, but weighting between-neighborhood effects by these metrics did not explain significantly more variation than in unweighted mixing between neighborhoods. This may be because when the force of infection is high in several neighborhoods simultaneously, the probability of contact (and hence transmission) is increased to most neighborhoods, thus diluting the role of more specific patterns of connectivity (similar idea to theoretical work showing that high rates of movement increase overall transmission [35]). However, because of the importance of the term for the interaction between mosquito density and cases, it is possible that a more direct measure of neighborhood connectivity (e.g. measurements of between-neighborhood human movement) would improve the predictive power of our model by making more accurate spatial predictions when transmission rates are lower.

### Generality of the vitoria model

Our analysis showed that the best model (as determined using Vitoria data) performed quite well at multiple tests of predictive power: forecasting future data in Vitoria as well as prediction of in- and out-of-sample data in two additional cities. This emphasizes that the general structure of the Vitoria model is a useful framework for quantifying different scales of spatial coupling in different cities. However, because the operational scale of vector control is the city block, using our model structure with block-level case and mosquito surveillance data will be most useful for directing operational work spatially.

We expected that the lag-time between mosquito density and human cases would approximate the virus life cycle (i.e., extrinsic incubation period + search time + intrinsic incubation period). While this was true for GV and SL (2 and 6 week lags), for Vitoria, the strongest signal was at a 13-week lag (although a strong signal was also observed at 4 weeks). The difference between cities in the most significant lag time between mosquito density and cases could be due to differences in the temporal patterns of vector control work (i.e., variable resources over time), the relative emphasis of different types of control (i.e., response-based versus prevention-based), or the total amount of resources available to conduct vector control (i.e., ability to respond to some versus many high-risk sites). We attempted to investigate these factors using vector control data from the 3 cities but we discovered that much of the control work was unrecorded. A study that includes a standardized method for recording the dates, times, location, type and amount of vector control - alongside MI-dengue surveillance - will be instrumental in interpreting the effects of control on the lag between mosquito density and human cases, and ultimately on reducing uncertainty on how to spatially prioritize vector control work.

The strong predictive ability of the case data alone shows that reasonable quantitative neighborhood-level predictions, especially with regards to the timing and magnitude of outbreaks, can be made from case notification data in the absence of mosquito surveillance data. Additionally, although results from Vitoria are based on almost 5 years of weekly data, similarly good fits and forecasts were possible in GV where less than 2 years of weekly data were available. However, the best forecasts were from models that included only space-time autocorrelation, instead of biological covariates (models not presented here). Thus, if the interest is in prediction for response planning, a non-mechanistic saturated model based on autocorrelation is likely to be the best approach. We did not present these models because our interest was in gaining an understanding of the relative role of biological factors and their spatial scales. Furthermore, the case data are often not available until about 6–8 weeks after diagnosis, which is why it is important to explore the utility of other data sources that may be available sooner.

## Conclusion

A mechanistic understanding of how mosquito density maps to disease transmission among humans is crucial for the development of quantitative tools that could guide spatial prioritization of vector control [10,35,37]. Despite the demonstrated efficacy of MI-dengue at preventing cases of dengue fever in several cities [16], our current work emphasizes that even further case reductions may be achieved if spatial prioritization occurred by additionally considering city-wide neighborhood connectivity – i.e., prioritizing highly connected areas with high mosquito density. As we did not find that readily available proxies of neighborhood connectivity explained spatial coupling, direct measures of city-wide connectivity (*e.g.,* space use by humans [13]) seem important for maximizing the preventative utility of mosquito surveillance data. Once these data are available, they can be used to identify which areas with high densities of mosquitos are most critical for targeting vector control in order to minimize transmission of dengue viruses among humans. A complimentary approach is to develop a spatially-explicit disease dynamic model that could be used to estimate city-wide connectivity, identify transmission hotspots and identify strategies of vector control that minimize city-wide cases. These are the goals of our ongoing research. Future research should also include city-wide MI-virus data as they become available. Ideally, case data should be collected at the block level, the operational unit, and serotype-specific case data are important for a better understanding of how to employ mosquito density data for spatial prioritization of vector control.

## Additional files

Additional file 1: Table S1. The “Traps” spreadsheet show the city-wide mosquito counts and number of traps monitored for each week in each city. The “Neighborhoods” spread sheet gives the population size and total area (km2) for each neighborhood in each city. Additional file 2: Figure S1. Model selection on scaling parameters for Vitoria. The covariate data describing between-neighborhood effects (Mjα, [Yjf(x)j]α and [ MjYjf(x)j]α) were scaled because these terms were much larger than those describing the within-neighborhood effects (Mi, Ii and Mi Ii). An initial attempt to fit the scaling parameters yielded lack of convergence, thus we conducted model selection on a range of pre-selected parameter values (α = 0.001, 0.01, 0.1, 0.5, 1, 2; indicated in the legend). Only single variable models were investigated (Model structure: log(yi,t) = Xj,tα + πi + log(Pi), where X is defined on the X-axis). (A) DIC (B) Spearman’s correlation between observed and model-predicted data. Only results from the best lags are presented for each scaling factor (selected from the analysis shown in Additional file 3: Figure S2). M = mosquito density, Y = reported cases, d = distance, E = economic value, D = density, i = focal neighborhood, j = all other neighborhoods (i≠j). Additional file 3: Figure S2. Model selection on covariate lags for Vitoria. Preliminary analyses showed that models with lower DIC’s were obtained when weekly data were averaged over 3-week windows. Thus, covariate data for all analyses were 3-week averages from the week indicated on the X-axis to two weeks in the future (i.e., 1 indicates an average of weeks 1–3). Left-hand plots indicate DIC for each single-variable model (structure: log(yi,t) = X + πi + log(Pi); where X represents the covariate in the figure legend), at each lag indicated on the X-axis. Right-hand plots display Spearman’s r for the same set of models. Only results from the best scaling factors (selected from the analysis shown in Additional file 2: Figure S1) are shown. (A) Mosquito-only covariates. (B) Case-only covariates. (C) Covariates with an interaction between mosquito density and cases. Black indicates within-neighborhood effects, red is nearest-neighbor between-neighborhood effects, blue is global between-neighborhood effects. Weighting terms are thin lines that are almost completely overlapping, showing that there was not much difference in the type of approximation used for weighting global connectivity. Additional file 4: Figure S3. Model selection on scaling parameters for GV. The covariate data describing between-neighborhood effects (Mjα, [Yjf(x)j]α and [ MjYjf(x)j]α) were scaled because these terms were much larger than those describing the within-neighborhood effects (Mi, Ii and Mi Ii). An initial attempt to fit the scaling parameters yielded lack of convergence, thus we conducted model selection on a range of pre-selected parameter values (α = 0.001, 0.01, 0.1, 0.5, 1, 2; indicated in the legend). Only single variable models were investigated (Model structure: log(yi,t) = Xj,tα + πi + log(Pi), where X is defined on the X-axis). (A) DIC (B) Spearman’s correlation between observed and model-predicted data. Only results from the best lags are presented for each scaling factor (selected from the analysis shown in Additional file 5: Figure S4). M = mosquito density, Y = reported cases, d = distance, C = number of commercial buildings, R = number of residences, D = density, i = focal neighborhood, j = all other neighborhoods (i≠j). Additional file 5: Figure S4. Model selection on covariate lags for GV. Preliminary analyses showed that models with lower DIC’s were obtained when weekly data were averaged over 3-week windows. Thus, covariate data for all analyses were 3-week averages from the week indicated on the X-axis to two weeks in the future (i.e., 1 indicates an average of weeks 1–3). Left-hand plots indicate DIC for each single-variable model (structure: log(yi,t) = X + πi + log(Pi); where X represents the covariate in the figure legend), at each lag indicated on the X-axis. Right-hand plots display Spearman’s r for the same set of models. Only results from the best scaling factors (selected from the analysis shown in Additional file 4: Figure S3) are shown. (A) Mosquito-only covariates. (B) Case-only covariates. (C) Covariates with an interaction between mosquito density and cases. Black indicates within-neighborhood effects, blue is global between-neighborhood effects. Weighting terms are thin lines that are almost completely overlapping in some cases, showing that there was not much difference in the type of approximation used for weighting global connectivity. Additional file 6: Figure S5. Model selection on scaling parameters for SL. The covariate data describing between-neighborhood effects (Mjα, [Yjf(x)j]α and [ MjYjf(x)j]α) were scaled because these terms were much larger than those describing the within-neighborhood effects (Mi, Ii and Mi Ii). An initial attempt to fit the scaling parameters yielded lack of convergence, thus we conducted model selection on a range of pre-selected parameter values (α = 0.001, 0.01, 0.1, 0.5, 1, 2; indicated in the legend). Only single variable models were investigated (Model structure: log(yi,t) = Xj,tα + πi + log(Pi), where X is defined on the X-axis). (A) DIC (B) Spearman’s correlation between observed and model-predicted data. Only results from the best lags are presented for each scaling factor (selected from the analysis shown in Additional file 7: Figure S6). M = mosquito density, Y = reported cases, d = distance, C = number of commercial buildings, R = number of residences, D = density, i = focal neighborhood, j = all other neighborhoods (i≠j). Additional file 7: Figure S6. Model selection on covariate lags for SL. Preliminary analyses showed that models with lower DIC’s were obtained when weekly data were averaged over 3-week windows. Thus, covariate data for all analyses were 3-week averages from the week indicated on the X-axis to two weeks in the future (i.e., 1 indicates an average of weeks 1–3). Left-hand plots indicate DIC for each single-variable model (structure: log(yi,t) = X + πi + log(Pi); where X represents the covariate in the figure legend), at each lag indicated on the X-axis. Right-hand plots display Spearman’s r for the same set of models. Only results from the best scaling factors (selected from the analysis shown in Additional file 6: Figure S5) are shown. (A) Mosquito-only covariates. (B) Case-only covariates. (C) Covariates with an interaction between mosquito density and cases. Black indicates within-neighborhood effects, blue is global between-neighborhood effects. Weighting terms are thin lines that are almost completely overlapping, showing that there was not much difference in the type of approximation used for weighting global connectivity. Additional file 8: Figure S7. Model selection on covariate lags for data aggregated to the city-wide scale. X-axes show the 3-week lag windows. Only a single-variable model with the mosquito density data was fit for each lag. Model structure: log(yt) = Mt; note that there are no neighborhood random effects or offset in this model because data from each time step are the total cases and mosquito density for the entire city. (A) DIC. (B) R2 (as in simple linear regression). Additional file 9: Figure S8. Predicted cases from the city-level mosquito density models. City-wide weekly cases are predicted from city-wide mosquito density data using a generalized linear model assuming a Poisson error structure and a log link. Parameter estimation was by INLA (same method used in the neighborhood-level models). Model selection was conducted on lags of mosquito density between 1 and 20 weeks prior to case reports. Mosquito density data were smoothed as three-week averages using a 1-week sliding window. (A) Lag of 1 to 3 weeks. (B) Lag of 13 to 15 weeks (shown to be the best by DIC and explained variation). Additional file 10: Figure S9. Performance of best models for GV (A) and SL (B). Spearman’s r is indicated for the city-level and neighborhood-level fits for both predictions from the fitted model and forecasts from the model (i.e., of data that were not used in model selection). Best models were: (A) Yi,t = β1Mi,t-2 + β2Yi,t-1 + β3Mi,t-4Yi,t-4 + β4∑jMj,t-12 + β5∑j(Yj,t-1Dj) 0.1 + β6∑j(Mj,t-1Yj,t-1Dj) 0.5 + log(Pi) + πi and (B) Yi,t = β1Mi,t-6 + β2Yi,t-1 + β3Mi,t-1Yi,t-1 + β4∑jMj,t-60.1 + β5∑j(Yj,t-1Rj) 0.001 + β6∑j(Mj,t-1 Yj,t-1Rj) 0.1 + log(Pi) + πi.
