# Supplementary figures and images for: Utility of mosquito surveillance data for spatial prioritization of vector control against dengue viruses in three Brazilian cities
Source: Parasit Vectors. 2015 Feb 15;8:98. doi: 10.1186/s13071-015-0659-y (PMC4335543; doi:10.1186/s13071-015-0659-y)

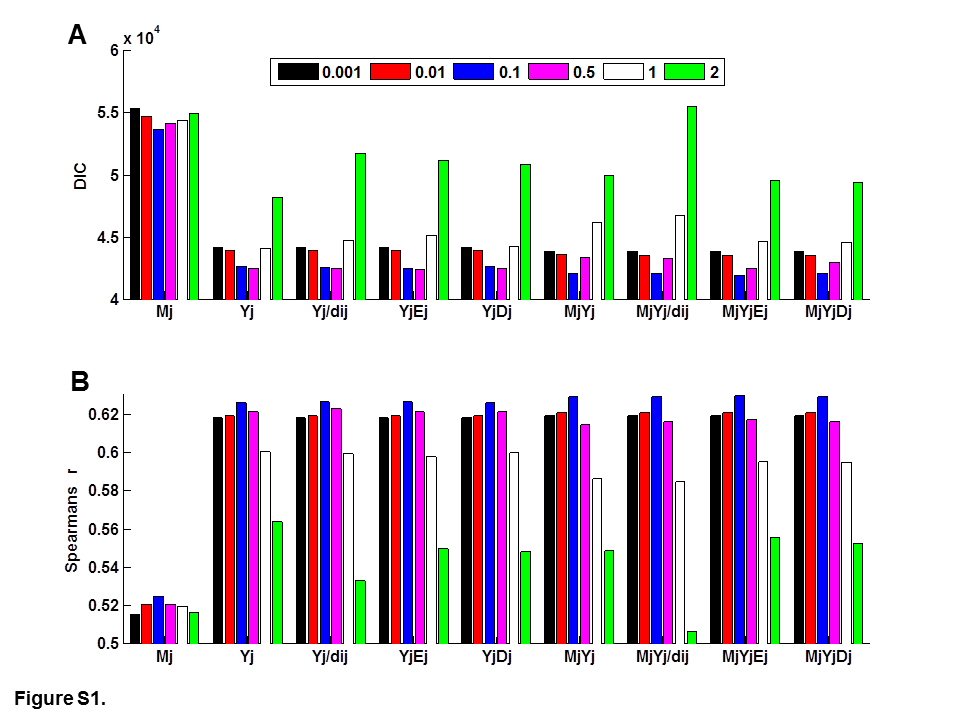

Supplement: Additional file 2: Figure S1. — Model selection on scaling parameters for Vitoria. The covariate data describing between-neighborhood effects (Mjα, [Yjf(x)j]α and [ MjYjf(x)j]α) were scaled because these terms were much larger than those describing the within-neighborhood effects (Mi, Ii and Mi Ii). An initial attempt to fit the scaling parameters yielded lack of convergence, thus we conducted model selection on a range of pre-selected parameter values (α = 0.001, 0.01, 0.1, 0.5, 1, 2; indicated in the legend). Only single variable models were investigated (Model structure: log(yi,t) = Xj,tα + πi + log(Pi), where X is defined on the X-axis). (A) DIC (B) Spearman’s correlation between observed and model-predicted data. Only results from the best lags are presented for each scaling factor (selected from the analysis shown in Additional file 3: Figure S2). M = mosquito density, Y = reported cases, d = distance, E = economic value, D = density, i = focal neighborhood, j = all other neighborhoods (i≠j). [file 13071_2015_659_MOESM2_ESM.tif]

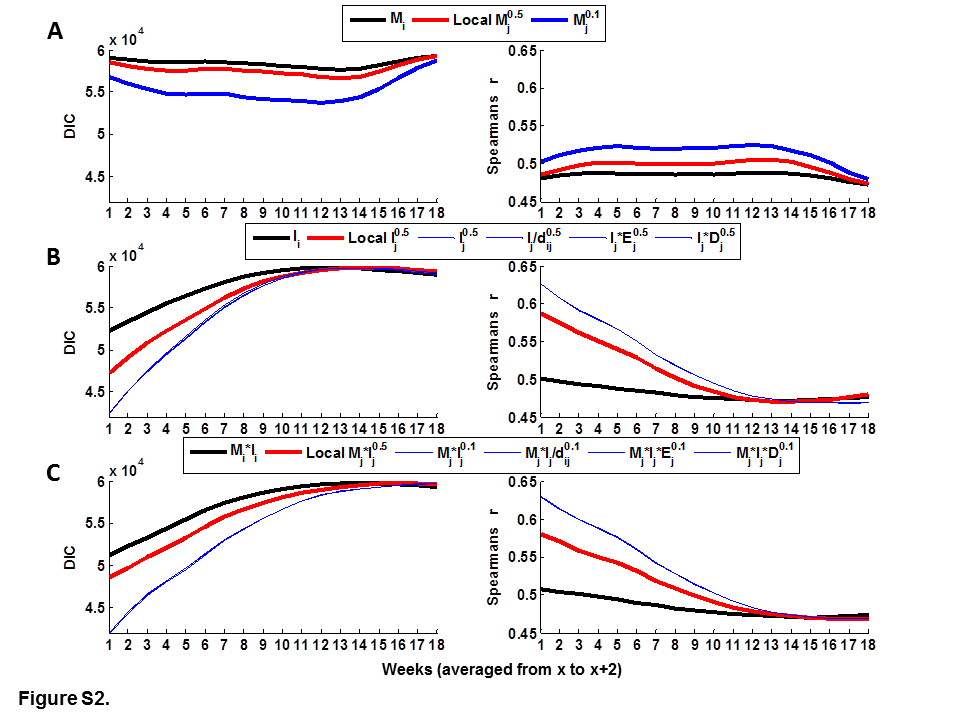

Supplement: Additional file 3: Figure S2. — Model selection on covariate lags for Vitoria. Preliminary analyses showed that models with lower DIC’s were obtained when weekly data were averaged over 3-week windows. Thus, covariate data for all analyses were 3-week averages from the week indicated on the X-axis to two weeks in the future (i.e., 1 indicates an average of weeks 1–3). Left-hand plots indicate DIC for each single-variable model (structure: log(yi,t) = X + πi + log(Pi); where X represents the covariate in the figure legend), at each lag indicated on the X-axis. Right-hand plots display Spearman’s r for the same set of models. Only results from the best scaling factors (selected from the analysis shown in Additional file 2: Figure S1) are shown. (A) Mosquito-only covariates. (B) Case-only covariates. (C) Covariates with an interaction between mosquito density and cases. Black indicates within-neighborhood effects, red is nearest-neighbor between-neighborhood effects, blue is global between-neighborhood effects. Weighting terms are thin lines that are almost completely overlapping, showing that there was not much difference in the type of approximation used for weighting global connectivity. [file 13071_2015_659_MOESM3_ESM.tif]

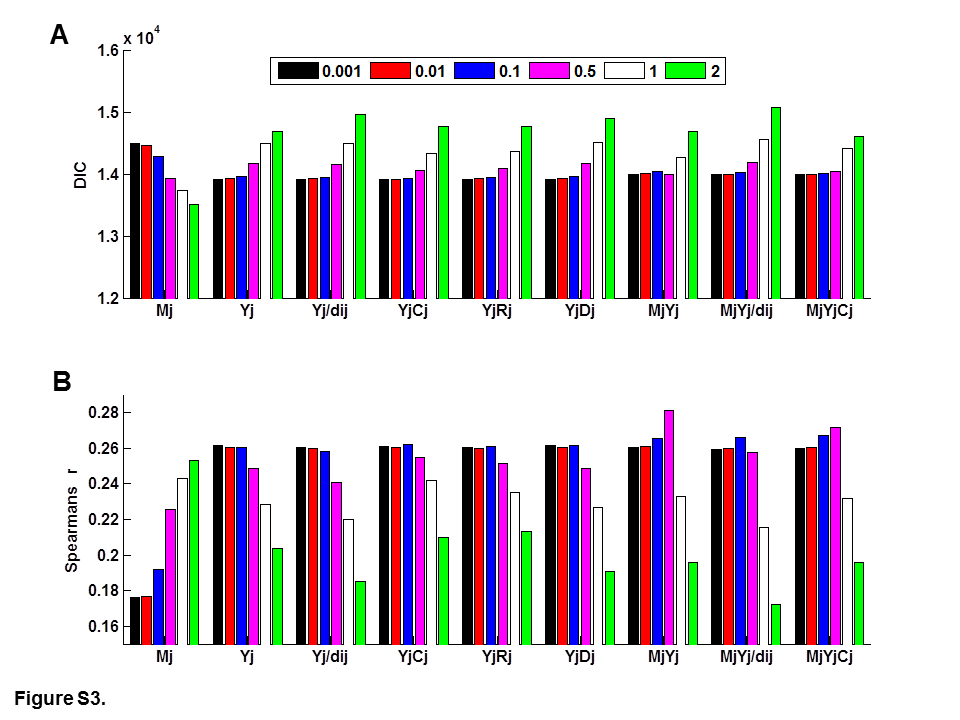

Supplement: Additional file 4: Figure S3. — Model selection on scaling parameters for GV. The covariate data describing between-neighborhood effects (Mjα, [Yjf(x)j]α and [ MjYjf(x)j]α) were scaled because these terms were much larger than those describing the within-neighborhood effects (Mi, Ii and Mi Ii). An initial attempt to fit the scaling parameters yielded lack of convergence, thus we conducted model selection on a range of pre-selected parameter values (α = 0.001, 0.01, 0.1, 0.5, 1, 2; indicated in the legend). Only single variable models were investigated (Model structure: log(yi,t) = Xj,tα + πi + log(Pi), where X is defined on the X-axis). (A) DIC (B) Spearman’s correlation between observed and model-predicted data. Only results from the best lags are presented for each scaling factor (selected from the analysis shown in Additional file 5: Figure S4). M = mosquito density, Y = reported cases, d = distance, C = number of commercial buildings, R = number of residences, D = density, i = focal neighborhood, j = all other neighborhoods (i≠j). [file 13071_2015_659_MOESM4_ESM.tif]

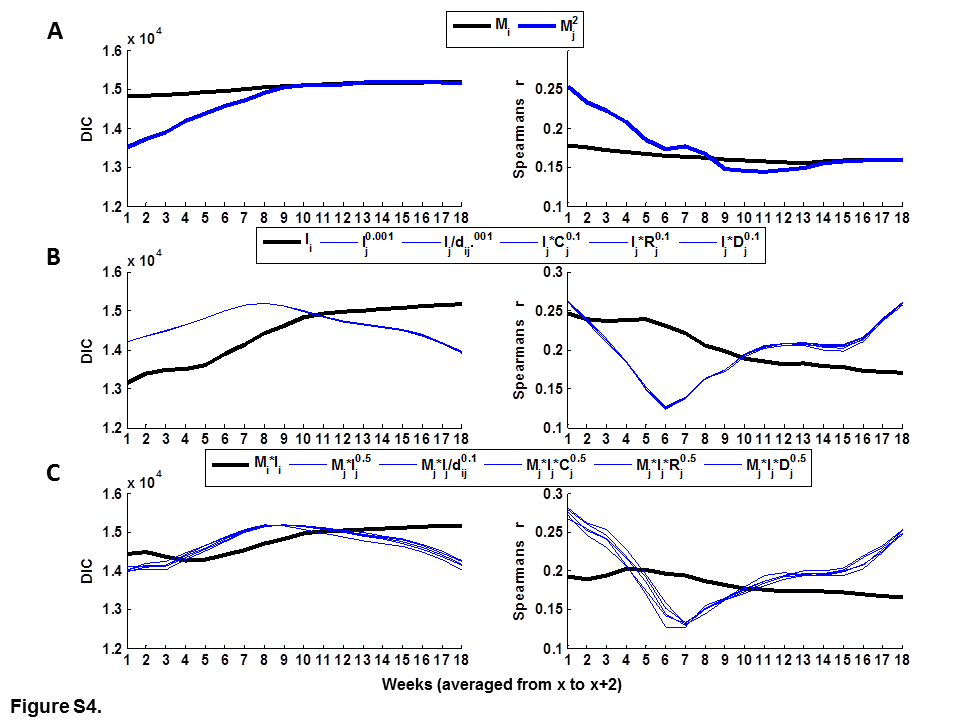

Supplement: Additional file 5: Figure S4. — Model selection on covariate lags for GV. Preliminary analyses showed that models with lower DIC’s were obtained when weekly data were averaged over 3-week windows. Thus, covariate data for all analyses were 3-week averages from the week indicated on the X-axis to two weeks in the future (i.e., 1 indicates an average of weeks 1–3). Left-hand plots indicate DIC for each single-variable model (structure: log(yi,t) = X + πi + log(Pi); where X represents the covariate in the figure legend), at each lag indicated on the X-axis. Right-hand plots display Spearman’s r for the same set of models. Only results from the best scaling factors (selected from the analysis shown in Additional file 4: Figure S3) are shown. (A) Mosquito-only covariates. (B) Case-only covariates. (C) Covariates with an interaction between mosquito density and cases. Black indicates within-neighborhood effects, blue is global between-neighborhood effects. Weighting terms are thin lines that are almost completely overlapping in some cases, showing that there was not much difference in the type of approximation used for weighting global connectivity. [file 13071_2015_659_MOESM5_ESM.tif]

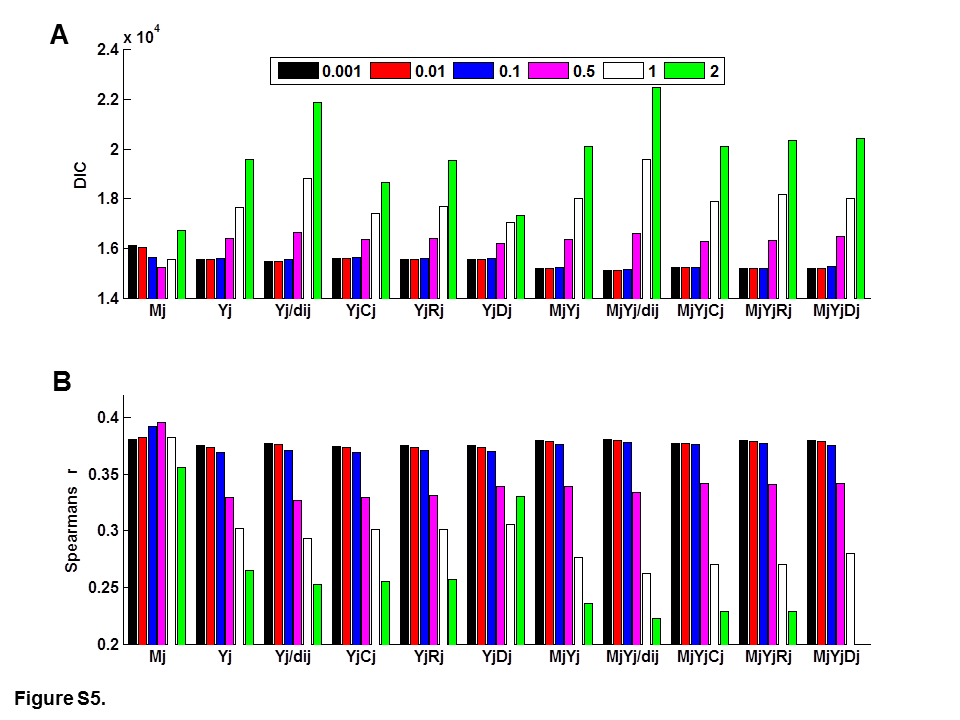

Supplement: Additional file 6: Figure S5. — Model selection on scaling parameters for SL. The covariate data describing between-neighborhood effects (Mjα, [Yjf(x)j]α and [ MjYjf(x)j]α) were scaled because these terms were much larger than those describing the within-neighborhood effects (Mi, Ii and Mi Ii). An initial attempt to fit the scaling parameters yielded lack of convergence, thus we conducted model selection on a range of pre-selected parameter values (α = 0.001, 0.01, 0.1, 0.5, 1, 2; indicated in the legend). Only single variable models were investigated (Model structure: log(yi,t) = Xj,tα + πi + log(Pi), where X is defined on the X-axis). (A) DIC (B) Spearman’s correlation between observed and model-predicted data. Only results from the best lags are presented for each scaling factor (selected from the analysis shown in Additional file 7: Figure S6). M = mosquito density, Y = reported cases, d = distance, C = number of commercial buildings, R = number of residences, D = density, i = focal neighborhood, j = all other neighborhoods (i≠j). [file 13071_2015_659_MOESM6_ESM.tif]

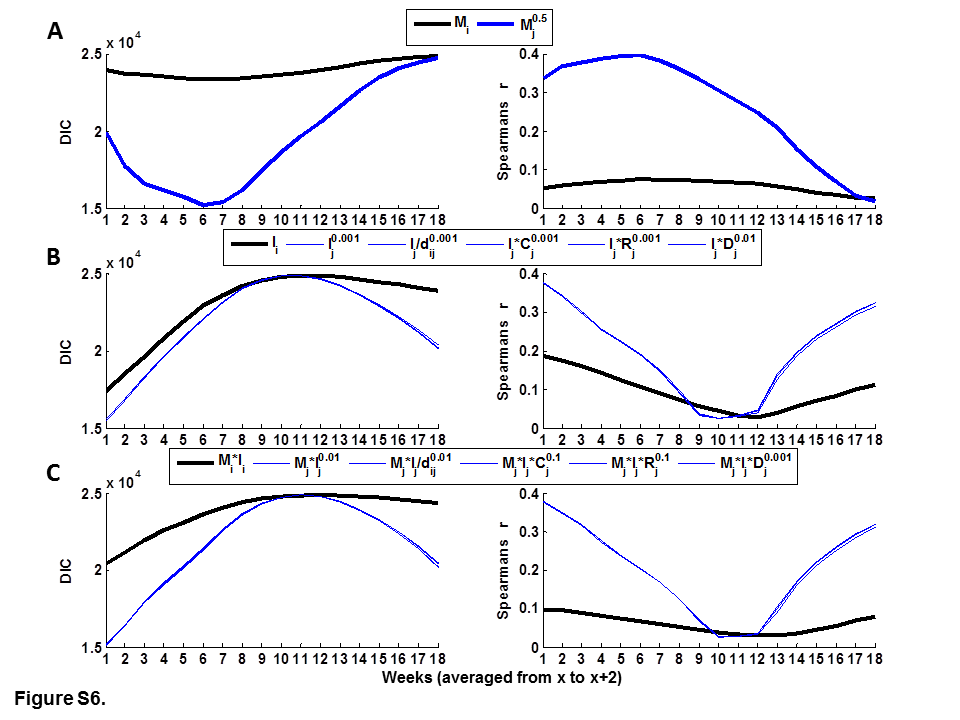

Supplement: Additional file 7: Figure S6. — Model selection on covariate lags for SL. Preliminary analyses showed that models with lower DIC’s were obtained when weekly data were averaged over 3-week windows. Thus, covariate data for all analyses were 3-week averages from the week indicated on the X-axis to two weeks in the future (i.e., 1 indicates an average of weeks 1–3). Left-hand plots indicate DIC for each single-variable model (structure: log(yi,t) = X + πi + log(Pi); where X represents the covariate in the figure legend), at each lag indicated on the X-axis. Right-hand plots display Spearman’s r for the same set of models. Only results from the best scaling factors (selected from the analysis shown in Additional file 6: Figure S5) are shown. (A) Mosquito-only covariates. (B) Case-only covariates. (C) Covariates with an interaction between mosquito density and cases. Black indicates within-neighborhood effects, blue is global between-neighborhood effects. Weighting terms are thin lines that are almost completely overlapping, showing that there was not much difference in the type of approximation used for weighting global connectivity. [file 13071_2015_659_MOESM7_ESM.tif]

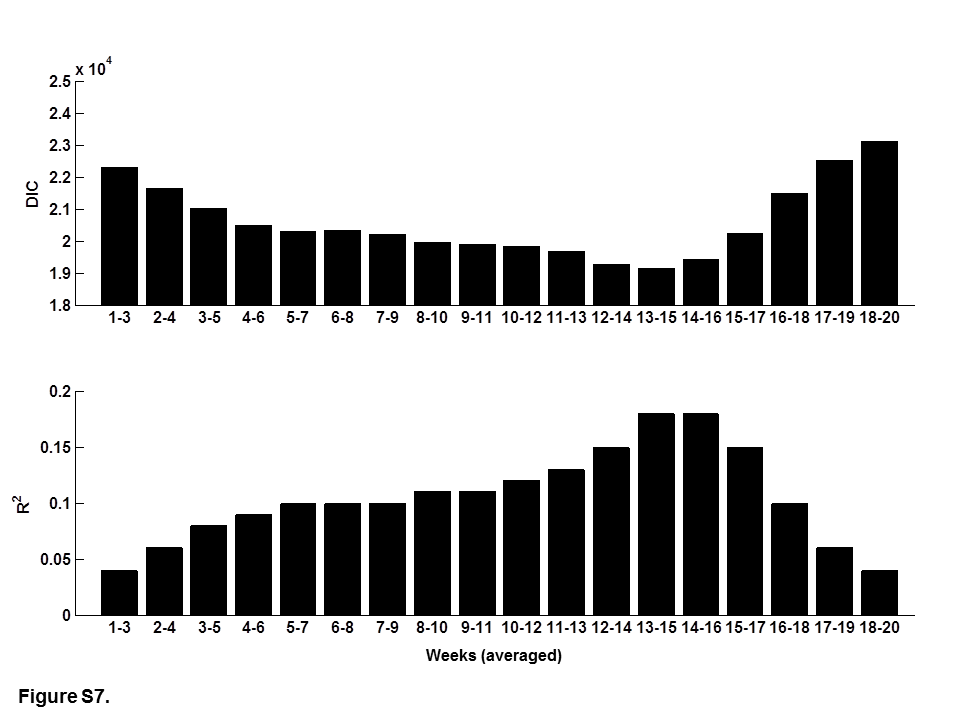

Supplement: Additional file 8: Figure S7. — Model selection on covariate lags for data aggregated to the city-wide scale. X-axes show the 3-week lag windows. Only a single-variable model with the mosquito density data was fit for each lag. Model structure: log(yt) = Mt; note that there are no neighborhood random effects or offset in this model because data from each time step are the total cases and mosquito density for the entire city. (A) DIC. (B) R2 (as in simple linear regression). [file 13071_2015_659_MOESM8_ESM.tif]

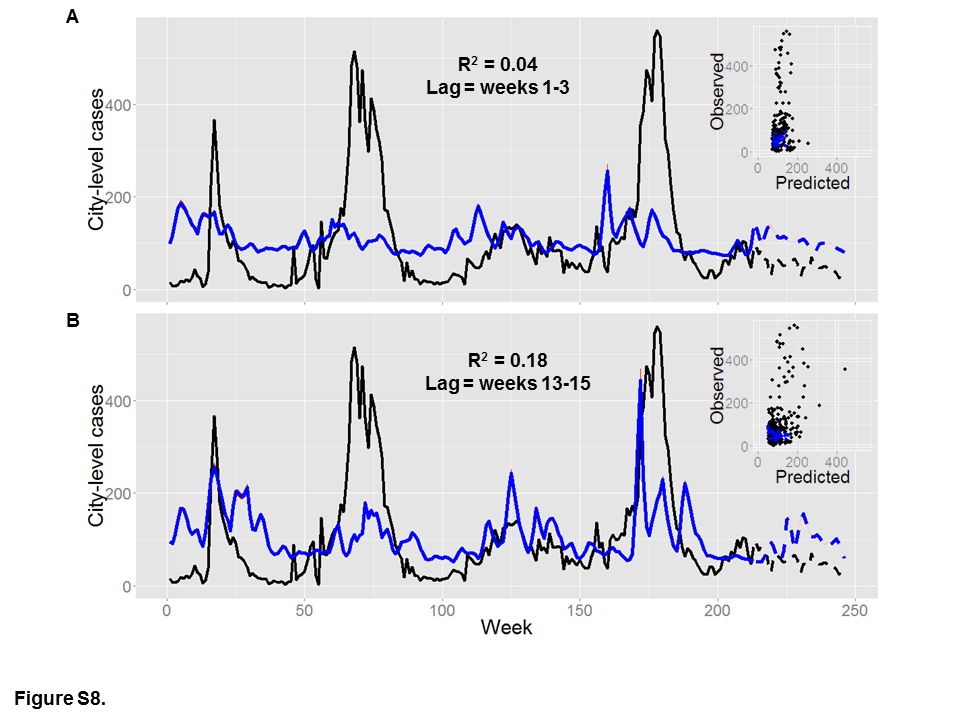

Supplement: Additional file 9: Figure S8. — Predicted cases from the city-level mosquito density models. City-wide weekly cases are predicted from city-wide mosquito density data using a generalized linear model assuming a Poisson error structure and a log link. Parameter estimation was by INLA (same method used in the neighborhood-level models). Model selection was conducted on lags of mosquito density between 1 and 20 weeks prior to case reports. Mosquito density data were smoothed as three-week averages using a 1-week sliding window. (A) Lag of 1 to 3 weeks. (B) Lag of 13 to 15 weeks (shown to be the best by DIC and explained variation). [file 13071_2015_659_MOESM9_ESM.tif]

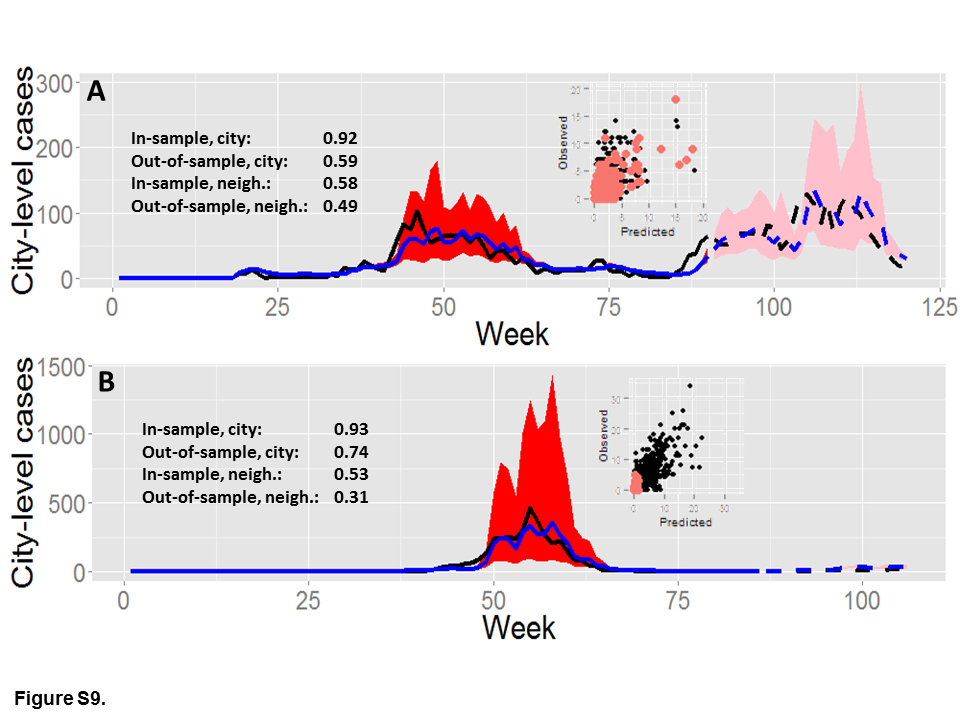

Supplement: Additional file 10: Figure S9. — Performance of best models for GV (A) and SL (B). Spearman’s r is indicated for the city-level and neighborhood-level fits for both predictions from the fitted model and forecasts from the model (i.e., of data that were not used in model selection). Best models were: (A) Yi,t = β1Mi,t-2 + β2Yi,t-1 + β3Mi,t-4Yi,t-4 + β4∑jMj,t-12 + β5∑j(Yj,t-1Dj) 0.1 + β6∑j(Mj,t-1Yj,t-1Dj) 0.5 + log(Pi) + πi and (B) Yi,t = β1Mi,t-6 + β2Yi,t-1 + β3Mi,t-1Yi,t-1 + β4∑jMj,t-60.1 + β5∑j(Yj,t-1Rj) 0.001 + β6∑j(Mj,t-1 Yj,t-1Rj) 0.1 + log(Pi) + πi. [file 13071_2015_659_MOESM10_ESM.tif]
